# Potential Role of the Triple Receptor Agonist Retatrutide in Antipsychotic-Associated Metabolic Dysfunction Among Clozapine- and Olanzapine-Treated Schizophrenia Spectrum Disorders: A Narrative Review and Theoretical Framework

**DOI:** 10.3390/biomedicines14091957

**Published:** 2026-08-31

**Authors:** Michael Mieczyslaw Kaczynski, Rina Maria Zureikat, Matthew Roman Kaczynski, Ryszard Sitarz, Hanna Karakuła-Juchnowicz

**Affiliations:** 1 Student Research Club at the 1st Clinic of Psychiatry, Psychotherapy, and Early Intervention, Department of Psychiatry, Medical University of Lublin, Głuska 1, 20-439 Lublin, Poland; rmzureikat@outlook.com (R.M.Z.); mattrkaczynski@gmail.com (M.R.K.); 2 Chair and Department of Psychiatry, Psychotherapy, and Early Intervention, Medical University of Lublin, Głuska 1, 20-439 Lublin, Poland; ryszard.sitarz@umlub.edu.pl (R.S.); hanna.karakula-juchnowicz@umlub.edu.pl (H.K.-J.)

**Keywords:** retatrutide, antipsychotic-associated metabolic dysfunction, antipsychotic-induced weight gain, schizophrenia spectrum disorders, clozapine, olanzapine, GLP-1 receptor agonists, incretin-based therapy, glucagon receptor agonism, drug–drug interactions

## Abstract

Antipsychotic-associated metabolic dysfunction contributes substantially to cardiometabolic morbidity and premature mortality in schizophrenia spectrum disorders, with the greatest burden among patients treated with clozapine and olanzapine. This review focuses on patients with schizophrenia spectrum disorders receiving these two agents, in whom the relevant psychiatric evidence is concentrated. Existing pharmacological interventions, including metformin and glucagon-like peptide-1 receptor agonists (GLP-1RAs), produce clinically meaningful but often incomplete benefits. Retatrutide (LY3437943) is a once-weekly triple agonist of the GLP-1, glucose-dependent insulinotropic polypeptide (GIP), and glucagon receptors that produced a mean body-weight reduction of 24.2% at 48 weeks under the efficacy estimand in a Phase 2 obesity trial; company-reported Phase 3 results from four trials, not yet peer-reviewed, describe reductions of up to 28.7%. This narrative review examines the rationale for investigating retatrutide in this setting, drawing on retatrutide trials in non-psychiatric populations, the pathophysiology of antipsychotic-induced weight gain, and emerging evidence for incretin-based therapies in antipsychotic-treated populations. The glucagon component is of particular mechanistic interest because it increases basal energy expenditure, a pathway not engaged by mono- or dual agonists; whether this could partially compensate for the reduced physical activity associated with antipsychotic-related sedation is untested and is presented here solely as a hypothesis. Indirect support comes from randomized trials and meta-analyses of GLP-1RAs in clozapine- or olanzapine-treated patients, which report reductions in body weight and glycemic impairment without evidence of worsening psychotic symptoms. No trial has evaluated retatrutide in an antipsychotic-treated population. Future studies should therefore characterize gastrointestinal tolerability, potential interaction with clozapine-associated gastrointestinal hypomotility, possible effects on clozapine and olanzapine exposure, adherence and feasibility, and the dysesthesia observed in retatrutide trials, which was dose-related in most but not all trials. Dedicated randomized controlled trials are needed to determine the efficacy and safety of retatrutide in patients with schizophrenia spectrum disorders receiving clozapine or olanzapine.

## 1. Introduction

People with schizophrenia experience a life-expectancy deficit of approximately 15 to 20 years relative to the general population [1], and that gap is widening rather than closing [2]; a 25-year community cohort study reported an all-cause standardized mortality ratio of 289 (95% CI 247 to 337) [3]. Although suicide carries the highest relative risk of any specific cause, cardiovascular disease accounts for a larger absolute share of excess mortality [1]. Multiple factors contribute to this disparity, including illness-related factors, lifestyle, disparities in healthcare access, and the adverse effects of psychotropic treatment [4]. In the broader severe mental illness (SMI) population, approximately 60% of adults are overweight or obese (pooled prevalence 60.1%, 95% CI 55.8 to 63.1) [5].

Antipsychotic-associated metabolic dysfunction encompasses weight gain, insulin resistance, dyslipidemia, hypertension, and related metabolic abnormalities occurring in antipsychotic-treated patients, against a background of both treatment-related and non-pharmacological risk factors. Metabolic syndrome was highly prevalent in the Clinical Antipsychotic Trials of Intervention Effectiveness (CATIE), which enrolled patients with chronic schizophrenia [6,7,8]. Among the 689 participants with complete fasting laboratory data, 40.9% met National Cholesterol Education Program (NCEP) criteria (51.6% of women and 36.0% of men), against 35.8% across the wider group of 1231, whose status could be classified with or without fasting values [6,8]. During Phase 1, metabolic syndrome prevalence increased among olanzapine-treated participants and decreased among those receiving ziprasidone [7]. Compared with an age-, gender-, and race/ethnicity-matched National Health and Nutrition Examination Survey (NHANES) III sample, CATIE men were 138% more likely and CATIE women 251% more likely to meet those criteria, and both differences persisted after adjustment for body mass index [8]. Clozapine and olanzapine carry among the greatest metabolic liabilities of currently used antipsychotics yet remain clinically essential in schizophrenia: clozapine reduces suicidal behavior in high-risk patients relative to olanzapine (hazard ratio 0.76, 95% CI 0.58 to 0.97) [9] and is the only agent with established efficacy in treatment-resistant illness [10]; olanzapine ranked third of 15 antipsychotics for overall efficacy in the largest comparative meta-analysis (standardized mean difference 0.59, 95% credible interval [CrI] 0.53 to 0.65), behind only clozapine and amisulpride [11].

Since discontinuing clozapine or olanzapine is rarely feasible in responders, pharmacological mitigation of their cardiometabolic sequelae is a priority. GLP-1 receptor agonists (GLP-1RAs) have emerged as potent anti-obesity agents; semaglutide 2.4 mg achieves approximately 14.9% body-weight reduction in general obesity [12] and tirzepatide, a dual GLP-1/GIP agonist, up to 20.9% [13]. Multiple randomized controlled trials (RCTs) establish proof-of-concept in antipsychotic-treated populations, including recent semaglutide trials in patients treated with second-generation antipsychotics (SGAs) and in clozapine-treated patients and exenatide in olanzapine-treated patients [14,15,16]; foundational evidence includes the landmark Larsen et al. liraglutide trial [17], the TAO and CODEX exenatide trials [18,19,20], and meta-analyses pooling up to 10 RCTs [21,22,23]. Real-world evidence suggests that weight-loss response to incretin therapy may vary according to antipsychotic exposure [24], although confounding by indication and other unmeasured factors limit causal interpretation. Whether metabolic response differs meaningfully by antipsychotic agent requires prospective study.

Retatrutide (LY3437943) is a once-weekly injectable triple agonist at the GLP-1, GIP, and glucagon receptors [25,26,27]. In Phase 2, it produced body-weight reductions of up to 24.2% at 48 weeks, compared with 14.9% for semaglutide 2.4 mg and 20.9% for tirzepatide in their respective pivotal trials, although these are separate trials rather than head-to-head comparisons [12,13,27]. Company-reported Phase 3 topline results, which have not yet undergone full peer review, describe reductions of up to 28.7% at 68 weeks in TRIUMPH-4 and 28.3% at 80 weeks in the pivotal TRIUMPH-1 obesity trial, with broad cardiometabolic improvement [28,29]. Two further Phase 3 trials reported topline results in July 2026: TRIUMPH-2, in type 2 diabetes with obesity or overweight, and TRIUMPH-3, in severe obesity with established cardiovascular disease, with weight reductions of up to 20.8% and 22.6%, respectively [30]. Together with retatrutide’s glucagon-mediated enhancement of basal energy expenditure, theorized to complement GLP-1- and GIP-driven appetite suppression, these findings provide a rationale for investigating retatrutide in antipsychotic-associated metabolic dysfunction, although they do not provide evidence of efficacy in this setting.

Against this background, this review examines whether retatrutide provides a sufficient mechanistic and empirical rationale to justify a dedicated study in antipsychotic-associated metabolic dysfunction. We restrict the principal scope to patients with schizophrenia spectrum disorders receiving clozapine or olanzapine, the population in which cardiometabolic liability is greatest, therapeutic substitution is rarely feasible, and the incretin evidence is concentrated. Where evidence is drawn from broader severe mental illness, general obesity, or type 2 diabetes populations, we identify it as such. GLP-1 mono-agonists in antipsychotic-treated and severe mental illness populations have themselves recently been reviewed [31,32]; to our knowledge, however, no review has examined triple GLP-1/GIP/glucagon receptor agonism, or retatrutide specifically, as a mechanistic strategy for antipsychotic-associated metabolic dysfunction.

No clinical study has evaluated retatrutide in a psychiatric or antipsychotic-treated population. The framework advanced here is therefore hypothesis-generating and relies on extrapolation from obesity and type 2 diabetes trials, together with indirect evidence from other incretin therapies in antipsychotic-treated patients. It is intended to inform future trial development, not current prescribing. This review is organized around three lines of evidence: (1) the pathophysiology and clinical burden of antipsychotic-associated metabolic dysfunction, with emphasis on clozapine and olanzapine; (2) the pharmacology and clinical evidence for retatrutide, interpreted alongside incretin studies in antipsychotic-treated populations; and (3) a mechanistic and future trial-development framework for triple receptor agonism. We additionally consider potential pharmacokinetic interactions, dysesthesia, gastrointestinal risk, body composition, and implementation feasibility.

## 2. Search Strategy and Selection Criteria

This narrative review was informed by searches of PubMed/MEDLINE, Embase, and ClinicalTrials.gov from 1 January 2020 to 6 August 2026. Trial-registry records and company-reported sources were re-checked on 23 August 2026 to confirm that the late-stage development status described here remained current. Targeted searching and manual reference-list screening also continued during revision, and a small number of additional sources identified in that way are cited where they are the principal source for a specific point. Google Scholar was used as a supplementary source to identify recently published records not indexed in the primary databases and to perform forward-citation searches of key studies. Reference lists of relevant reviews and primary studies were screened manually, and foundational publications from before 2020 were included when they remained the principal source for a topic relevant to this review.

The primary search combined intervention-, population-, and outcome-related concepts using the following terms: (“retatrutide” OR “LY3437943” OR “triple receptor agonist” OR “GLP-1 receptor agonist” OR “semaglutide” OR “tirzepatide” OR “liraglutide” OR “exenatide” OR “incretin”) AND (“antipsychotic” OR “clozapine” OR “olanzapine” OR “schizophrenia” OR “severe mental illness” OR “antipsychotic-induced weight gain” OR “antipsychotic-associated metabolic dysfunction” OR “metabolic syndrome”). Additional targeted searches addressed pharmacokinetic interactions, therapeutic drug monitoring, gastrointestinal hypomotility, dysesthesia, body composition, lean-mass preservation, sarcopenic risk, glucagon and GIP receptor physiology, energy expenditure and physical activity, eating-disorder pathology, psychiatric safety and suicidality pharmacovigilance, medication adherence, renal and hepatic outcomes, metabolic monitoring guidance, and established pharmacological interventions for antipsychotic-induced weight gain.

The primary search was designed to capture the intersection of incretin-based therapy and antipsychotic treatment. Evidence concerning antipsychotic metabolic pathophysiology, specific safety domains, and foundational epidemiology was identified through these targeted searches and through manual reference-list screening rather than through the primary string.

Sources were considered for inclusion when they reported clinical or mechanistic evidence relevant to incretin-based therapies, antipsychotic-associated metabolic dysfunction, or the safety, pharmacokinetic, gastrointestinal, neurological, nutritional, or implementation issues addressed in this review. Peer-reviewed, English-language human studies were prioritized. Preclinical studies were included only when they provided mechanistic information that was not otherwise available from human evidence. Trial-registry records, conference presentations, and company-reported topline results were included where necessary to describe ongoing or recently completed retatrutide trials and were identified separately from peer-reviewed evidence. Sources not relevant to the clinical, mechanistic, or translational domains of the review were not retained.

Sources were classified as randomized trials, systematic reviews or meta-analyses, observational studies, case reports, preclinical or mechanistic studies, trial-registry records, conference presentations, or company-reported topline results. TRIUMPH-1, TRIUMPH-2, TRIUMPH-3, and TRIUMPH-4 were classified as preliminary and pending full peer-reviewed publication, whereas TRANSCEND-T2D-1 was classified as peer-reviewed Phase 3 evidence. Because this was a narrative rather than systematic review, study selection was relevance-guided, screening was not conducted in duplicate, and no formal risk-of-bias assessment or quantitative synthesis was undertaken.

Terminology is applied consistently throughout. “Antipsychotic-associated metabolic dysfunction” is used as an umbrella term for adverse metabolic abnormalities occurring in antipsychotic-treated patients. The term “associated” is deliberate, because metabolic risk in schizophrenia spectrum disorders reflects pharmacological effects alongside dietary, physical-activity, smoking-related, illness-related, and other factors, and the pharmacological contribution cannot be assumed to account for all of it. “Antipsychotic-induced weight gain” is reserved specifically for weight change, where a causal pharmacological contribution is well established. “Metabolic syndrome” is used only when the cited study applied formal diagnostic criteria, such as those of the National Cholesterol Education Program. Terms including obesity, prediabetes, dyslipidemia, and type 2 diabetes are likewise used according to the definitions applied in the source study.

## 3. Pathophysiology of Antipsychotic-Associated Metabolic Dysfunction

### 3.1. Receptor-Mediated Mechanisms of Antipsychotic Weight Gain

Antipsychotic-induced weight gain (AIWG) is strongly linked to antagonism at histamine H1 and serotonin 5-HT2C receptors, with additional contributions from dopamine D2 receptor signaling [33,34,35,36]. Mechanistically, H1 blockade activates hypothalamic AMP-activated protein kinase (AMPK), promoting hyperphagia [35,36], whereas 5-HT2C antagonism attenuates melanocortinergic satiety signaling via reduced pro-opiomelanocortin (POMC) activation in the arcuate nucleus [33,34]. The resulting hyperphagia is most pronounced with clozapine and olanzapine, which bind both receptors with high affinity [33,35].

Appetite dysregulation is central to AIWG: Huang et al. found that 77.4% of 31 drug-naive, first-episode patients developed increased appetite over 12 weeks of olanzapine treatment; increased appetite mediated 66.2% of subsequent weight gain, and patients with increased appetite gained 9.1 kg compared with 3.9 kg among those without [37]. This is consistent with evidence that adverse cardiometabolic changes can emerge within the first weeks of antipsychotic exposure [38,39].

Beyond appetite dysregulation, SGAs can exert direct effects on peripheral metabolic pathways. In healthy volunteers given olanzapine, aripiprazole, or placebo for nine days under controlled inpatient conditions, both antipsychotics induced insulin resistance in the absence of weight gain or psychiatric illness; only olanzapine additionally raised postprandial insulin, GLP-1, and glucagon concentrations [40]. Clozapine has also been associated with impaired peripheral glucose metabolism through mechanisms that may include altered insulin receptor signaling [41]. These treatment-related effects occur against a background of pre-existing metabolic vulnerability associated with genetic, environmental, and illness-related factors [42]. In one study, more than 15% of first-episode, drug-naive patients had impaired fasting glucose tolerance compared with none of the anthropometrically matched healthy controls; patients also had higher fasting insulin concentrations and greater insulin resistance [43]. A population-based cohort further reported a 3.07-fold adjusted risk of diabetes (95% CI 1.71 to 5.41) among antipsychotic-naive patients with schizophrenia, increasing to 3.64-fold (1.95 to 6.82) after antipsychotic initiation [44].

Clozapine’s metabolic effects may also extend beyond body-weight change. In obese mice, Chang et al. reported worsened glucose intolerance, hepatic steatosis, kidney damage, and retinal injury [45]. These preclinical findings suggest that clozapine-related metabolic effects may involve multiple organ systems, although their relevance to human antipsychotic-associated metabolic dysfunction requires clinical confirmation.

A further line of evidence concerns endogenous incretin signaling itself. Klemettilä et al. found that in male clozapine-treated patients, endogenous GLP-1 levels correlated with BMI (r = 0.34) and with weight gain; the authors interpreted this finding as a possible compensatory but insufficient incretin response to clozapine-associated metabolic stress [46]. Given the modest association and its restriction to one subgroup, this finding is hypothesis-generating and does not establish inadequate endogenous incretin signaling. It nevertheless provides a tentative mechanistic rationale for exogenous GLP-1 receptor agonism.

### 3.2. The CATIE Legacy: Metabolic Outcomes in a Pragmatic Effectiveness Trial

The CATIE trial remains among the most comprehensive prospective datasets on antipsychotic-associated metabolic dysfunction [6,7,47]. During Phase 1, mean monthly weight changes were +2.0 lb with olanzapine, +0.5 with quetiapine, +0.4 with risperidone, −0.2 with perphenazine, and −0.3 with ziprasidone; 30% of olanzapine-treated patients gained more than 7% of baseline body weight, against 7% to 16% in the other four treatment groups [47]. At baseline, metabolic syndrome prevalence was 40.9% among the 689 participants with complete fasting laboratory data and 35.8% across the 1231 participants whose status could be classified, far exceeding age-, gender-, and race/ethnicity-matched general-population estimates [6,8]. Separately, only 110 of 915 patients with dyslipidemia (12.0%) were receiving lipid-lowering therapy [48].

### 3.3. Metabolic Monitoring, Treatment Adherence, and Clinical Implications

Adherence to recommended metabolic monitoring remains suboptimal. Mitchell et al. reported glucose monitoring in 44.3% and cholesterol monitoring in 41.5% of antipsychotic-treated patients, while lipids and glycosylated hemoglobin were monitored in fewer than 25% [49]. This monitoring gap is clinically important given the excess cardiometabolic mortality in schizophrenia [1,2,3] and underscores the unmet clinical need this review addresses.

Weight gain is also an important contributor to medication non-adherence. Patients have been reported to take, on average, 58% of prescribed antipsychotic doses (range 24% to 90%) [50]. Across studies, non-adherence affects approximately 40% to 50% of patients with schizophrenia [51], with one report estimating that 48% are non-adherent within the first year of treatment [52]. Olanzapine was associated with 3.32-fold higher odds of non-adherence or discontinuation than antipsychotics of lower weight-gain propensity (95% CI 2.32 to 4.74, *p* < 0.00001), and agents of moderate liability with 2.25-fold higher odds (95% CI 1.31 to 3.87, *p* = 0.003) [53]. In CATIE, discontinuation attributed to weight gain or metabolic effects was more frequent with olanzapine than with any of the other four study drugs (9% vs. 1% to 4%, *p* < 0.001) [47], a pattern also emphasized in reviews of antipsychotic weight gain and adherence [54]. Obesity has been associated with more than a two-fold increase in self-reported non-adherence (odds ratio 2.5, 95% CI 1.1 to 5.5) [55]. Weight gain of at least 7% was also associated with antipsychotic switching within 180 days (odds ratio 1.60, *p* = 0.003) [56], a paradox reflecting that early weight gain marks consistent medication-taking even as the metabolic burden eventually motivates change. Because non-adherence is associated with relapse, greater healthcare use, and higher costs [51,54], whether effective weight management improves treatment continuity should be evaluated directly in future studies.

## 4. Comparative Metabolic Risk Profiles of Selected Antipsychotics

Table 1 summarizes the comparative metabolic profiles of selected antipsychotics. Agents were selected to include clozapine and olanzapine, the two antipsychotics of primary interest, together with commonly used comparator agents spanning the range from minimal to very high metabolic liability. Placebo-adjusted weight changes are taken from a single network meta-analysis; the qualitative risk classifications and clinical notes are author-derived from randomized, observational, and CATIE evidence [7,10,11,47,48,57,58,59,60,61,62,63,64,65,66,67,68].

Across comparative analyses, clozapine and olanzapine are consistently identified among the antipsychotics with the greatest metabolic liability. In a 10-year naturalistic study of 96 clozapine-treated patients, Henderson et al. reported a Kaplan–Meier estimate of approximately 43% for new-onset diabetes mellitus and an estimated 10-year cardiovascular mortality rate of 9% [69]. These findings illustrate the substantial long-term metabolic burden associated with clozapine treatment and the need for effective metabolic-risk mitigation.

Metabolic liability is not uniformly dose-dependent. In a prospective cohort of 392 olanzapine-treated patients, doses above 10 mg/day were associated with greater odds of early weight gain, defined as an increase of at least 5% within the first month (odds ratio 2.15, 95% CI 1.57 to 2.97), whereas weight change was not associated with dose when this was analyzed as a continuous variable [65,70]. By contrast, Piras et al. [71] reported a dose-dependent association for clozapine. Each 100 mg/day increment in clozapine dose was associated with an additional 0.48% increase in baseline body weight over one year; the dose–weight association emerged after approximately three months of treatment [71]. CYP1A2 variability and smoking status are discussed in Section 9.3.

## 5. Retatrutide: Mechanism of Action and Pharmacological Rationale

### 5.1. Triple Receptor Agonism: A Mechanistic Overview

**Figure 1:**
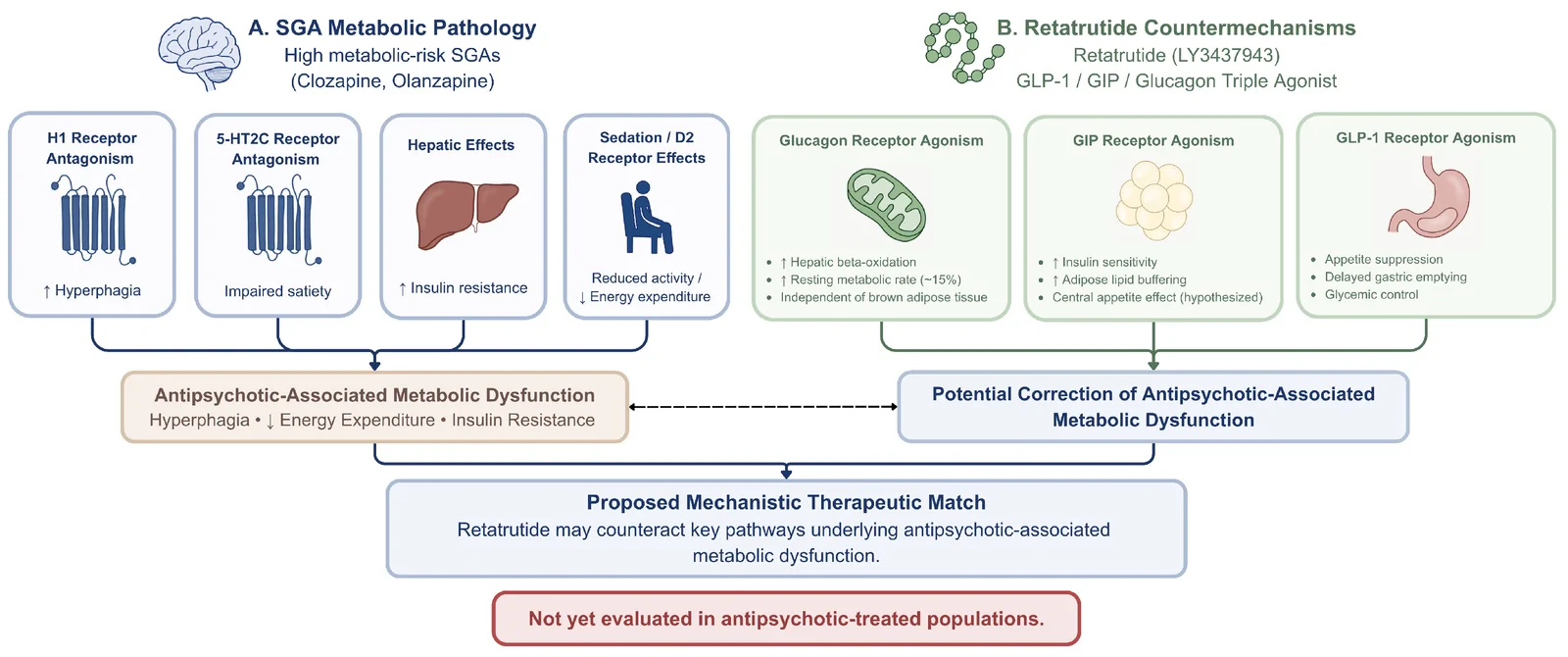
Proposed mechanistic rationale for retatrutide (LY3437943) in antipsychotic-associated metabolic dysfunction. The figure sets out a hypothesized mechanism by which retatrutide may counteract key pathways underlying antipsychotic-associated metabolic dysfunction. Retatrutide has not been studied in psychiatric or antipsychotic-treated populations, and every element of Panel (**B**) is extrapolated from obesity and diabetes data rather than demonstrated in this context. (**A**) illustrates the key pathways by which high metabolic-risk second-generation antipsychotics, particularly clozapine and olanzapine, drive weight gain and metabolic dysfunction: H1 receptor antagonism activates hypothalamic AMPK and promotes hyperphagia [35,36]; 5-HT2C receptor antagonism attenuates melanocortinergic satiety signaling through reduced POMC activation in the arcuate nucleus [33,34]; hepatic and peripheral effects contribute to insulin resistance independently of weight gain [40,41]; and sedation with D2-related effects contributes to reduced physical activity, which is markedly lower in this population than in matched controls and is associated with antipsychotic use, lowering the activity-related component of energy expenditure [79]. Together, these converge on the clinical phenotype of antipsychotic-associated metabolic dysfunction. (**B**) depicts the three complementary receptor-mediated mechanisms of retatrutide: glucagon receptor agonism, which acts principally on the liver to stimulate beta-oxidation [77] and raises resting metabolic rate by approximately 15% independently of brown adipose tissue activation [78]; GIP receptor agonism, which improves systemic insulin sensitivity and the lipid-buffering capacity of white adipose tissue and is hypothesized to contribute centrally to appetite suppression [75]; and GLP-1 receptor agonism, which suppresses appetite, delays gastric emptying, and improves glycemic control [73,74]. The glucagon receptor component is unique among current incretin-based therapies and is hypothesized to contribute additional basal energy expenditure; whether this offsets the reduced physical-activity energy expenditure associated with antipsychotic-induced sedation is untested, is not addressed by any available preclinical data, and is presented here as a mechanistic hypothesis rather than an established effect. The summary panel below the two arms states the proposition the figure illustrates; it is not a reported finding. Solid arrows indicate the direction of the proposed pathways. The dashed bidirectional arrow denotes a hypothesized correspondence between the two panels rather than a demonstrated effect. Upward and downward arrows within boxes denote increase and decrease.

Retatrutide (LY3437943) is a single-molecule peptide that simultaneously activates the GLP-1, GIP, and glucagon receptors [25] (Figure 1), distinguishing it mechanistically from the GLP-1 mono-agonist semaglutide and the GLP-1/GIP dual agonist tirzepatide [25,26,27,72].

Among its three targets, the GLP-1 component suppresses appetite via central satiety pathways, delays gastric emptying, and enhances glucose-dependent insulin secretion [73,74]. The GIP component is associated with improved systemic insulin sensitivity and enhances the lipid-buffering capacity of white adipose tissue [75]; a central contribution to appetite or weight regulation has also been proposed, although its magnitude and mechanism remain incompletely defined. The therapeutic implications of GIP-mediated lipogenic signaling remain debated, particularly because both GIP receptor agonism and antagonism reduce body weight in preclinical models [76]. The glucagon receptor component distinguishes retatrutide from semaglutide and tirzepatide. Glucagon signaling acts principally on the liver, where it stimulates beta-oxidation and may reduce hepatic lipid accumulation and secretion; effects on adipose tissue lipolysis are established in rodent adipocytes but remain controversial in humans [77]. Acute glucagon administration can increase resting energy expenditure independently of brown adipose tissue activation: in healthy adults, glucagon infusion increased resting metabolic rate by approximately 15%, matching the rise produced by cold exposure, without measurable brown adipose tissue activation [78]. Respiratory quotient also increased during glucagon infusion, which investigators attributed to increased carbohydrate oxidation; this whole-body substrate measure does not directly quantify hepatocyte-level beta-oxidation. The observed effect is therefore on the basal metabolic component of total energy expenditure, that is, on resting metabolic rate, rather than on arousal, motivation, or volitional physical activity. Low physical activity has been documented in the broader severe mental illness population: across 69 studies including 35,682 participants, 91.8% of whom were prescribed antipsychotics, the pooled sample spent a mean of 476 min per day sedentary during waking hours and engaged in 38 min of moderate or vigorous physical activity, both significantly lower than in age- and gender-matched controls. Antipsychotic use was among the factors associated with lower physical activity [79]. No preclinical or clinical data of which we are aware address whether glucagon receptor agonism can offset centrally mediated sedation. Accordingly, this remains an untested hypothesis rather than an established mechanism. Future trials could address this directly by pairing indirect calorimetry with objective actigraphy to distinguish resting from activity-related energy expenditure.

A related question is the extent to which incretin-mediated satiety signaling remains functional in the presence of ongoing H1 and 5-HT2C receptor blockade, given the contribution of these pathways to antipsychotic-induced hyperphagia (Section 3.1). The relationship appears to be one of partial convergence rather than full independence. A substantial component of GLP-1 receptor agonist action is exerted through hindbrain structures, including the nucleus tractus solitarius and the area postrema, together with vagal afferent signaling [73,74]. Because these pathways are not themselves targets of H1 or 5-HT2C receptor antagonism, they provide a plausible route through which GLP-1-mediated satiety signaling could remain operative during antipsychotic treatment. At the hypothalamic level, however, the pathways overlap: 5-HT2C-mediated satiety acts in part through activation of arcuate POMC neurons [33,34], and GLP-1 signaling engages the same population. A further point of mechanistic interaction may occur at hypothalamic AMPK: H1 antagonism promotes hyperphagia through AMPK activation [35,36], whereas incretin signaling has been associated with opposing effects on this pathway. These observations provide a mechanistic context for the clinical finding that GLP-1 receptor agonists retain efficacy in clozapine- and olanzapine-treated patients [14,15,17]. Whether overlap at hypothalamic pathways modifies the magnitude of response cannot be determined from existing data. The GIP component warrants separate comment, since it is less well characterized in this respect. The GIP receptor is distinct from the histaminergic, serotonergic, and dopaminergic receptors through which clozapine and olanzapine act [33,34]; GIP receptor signaling is therefore not directly blocked by H1, 5-HT2C, or D2 receptor antagonism, although its functional contribution during antipsychotic treatment has not been established. Its central contribution to satiety is, however, less clearly defined than that of GLP-1, and the two components should not be assumed to act equivalently in this population. These considerations remain mechanistic interpretations rather than demonstrated effects; no study has directly compared incretin-mediated satiety signaling in the presence vs. absence of antipsychotic receptor blockade.

An additional theoretical concern relates to body composition and the preservation of lean mass. Glucagon receptor signaling promotes hepatic amino acid uptake and ureagenesis, creating a mechanistic concern that sustained glucagon receptor agonism could contribute to lean-mass loss. A disproportionality analysis of FDA adverse-event reports has identified a muscle-atrophy signal with GLP-1 receptor agonists, which its authors frame as signal detection rather than evidence of causality [80]. Body-composition reviews estimate that roughly 20 to 40% of GLP-1RA-associated weight loss may be attributable to reductions in lean mass [81], although a more recent synthesis places the proportion at 20 to 30% and reports it as largely stable across trials [82]. Two caveats limit direct extrapolation to retatrutide. First, these estimates derive predominantly from GLP-1 receptor agonist and dual agonist studies; whether the addition of glucagon receptor agonism alters the proportion of lean-mass loss has not been established. Second, absolute lean-mass reduction could be clinically relevant even if the proportion of weight loss as lean mass is similar to that observed with other incretin therapies, because absolute loss tends to scale with total weight loss and the reductions reported with triple agonism are larger.

These considerations may be particularly relevant in schizophrenia spectrum disorders, given the low physical activity documented across schizophrenia and broader severe mental illness populations [79] and evidence of elevated sarcopenic risk. In what its authors describe as the first study to examine this, possible sarcopenia, defined as low muscle strength without confirmed loss of muscle mass, was identified in 26 of 72 patients with schizophrenia (36.1%), while confirmed and severe sarcopenia were absent [83]. An important interpretive caveat also applies: reductions in lean mass identified by imaging should not be equated with sarcopenia in the absence of demonstrated functional impairment, since some loss of lean tissue reflects physiological adaptation to negative energy balance rather than clinically meaningful muscle dysfunction [84]. The body-composition consequences of substantial weight loss in this population may therefore differ from those observed in general-obesity cohorts, and findings from the latter should not be assumed to transfer. Future trials in clozapine- or olanzapine-treated patients should therefore assess body composition directly by dual-energy X-ray absorptiometry (DXA) or bioelectrical impedance analysis rather than relying on body weight alone and should assess muscle function alongside muscle mass. Protocolized resistance exercise and a prespecified minimum protein intake should be considered standard co-interventions rather than optional adjuncts. These proposals are developed further in Section 9.7 and Section 10.3. Retatrutide may also affect circulating lipid regulators. Wen et al. reported dose-dependent reductions in circulating ANGPTL3/8, which paralleled improvements in triglycerides and LDL-cholesterol [85].

Preclinical evidence also suggests a CNS-dependent component of glucagon-mediated weight regulation. In mice, hepatic glucagon receptor signaling increases circulating fibroblast growth factor 21, and neuronal deletion of its coreceptor β-Klotho attenuates glucagon-receptor-mediated weight loss while preserving the lipid effects [86]. Whether retatrutide’s glucagon component produces clinically meaningful CNS effects in humans, or whether those effects are modified by antipsychotic receptor blockade, remains unknown.

### 5.2. Comparative Pharmacological Profile

No head-to-head trial has compared semaglutide, tirzepatide, and retatrutide. TRIUMPH-5, a Phase 3 randomized, double-blind trial comparing retatrutide with tirzepatide in 800 adults with obesity, has an estimated completion date of December 2026 and has not reported [87]. For descriptive context only, separate obesity trials reported mean body-weight reductions of 14.9% with semaglutide in STEP 1 [12], 20.9% with tirzepatide in SURMOUNT-1 [13], and 24.2% with retatrutide at 48 weeks in Phase 2 [27]; company-reported Phase 3 data describe reductions of up to 28.3% at 80 weeks [29]. These estimates derive from separate trials differing in population, entry criteria, dose, treatment duration, missing-data approach, and statistical estimand, and therefore should not be interpreted as evidence of comparative superiority. An abstract-only network meta-analysis by Salhab et al. reported an indirect estimate favoring retatrutide over tirzepatide for weight reduction, accompanied by a higher adverse-event rate [88]. Table 2 provides a descriptive comparison of receptor targets [89] and key trial characteristics.

## 6. Emerging Evidence for Incretin Therapies in Antipsychotic-Treated Populations

**Figure 2:**
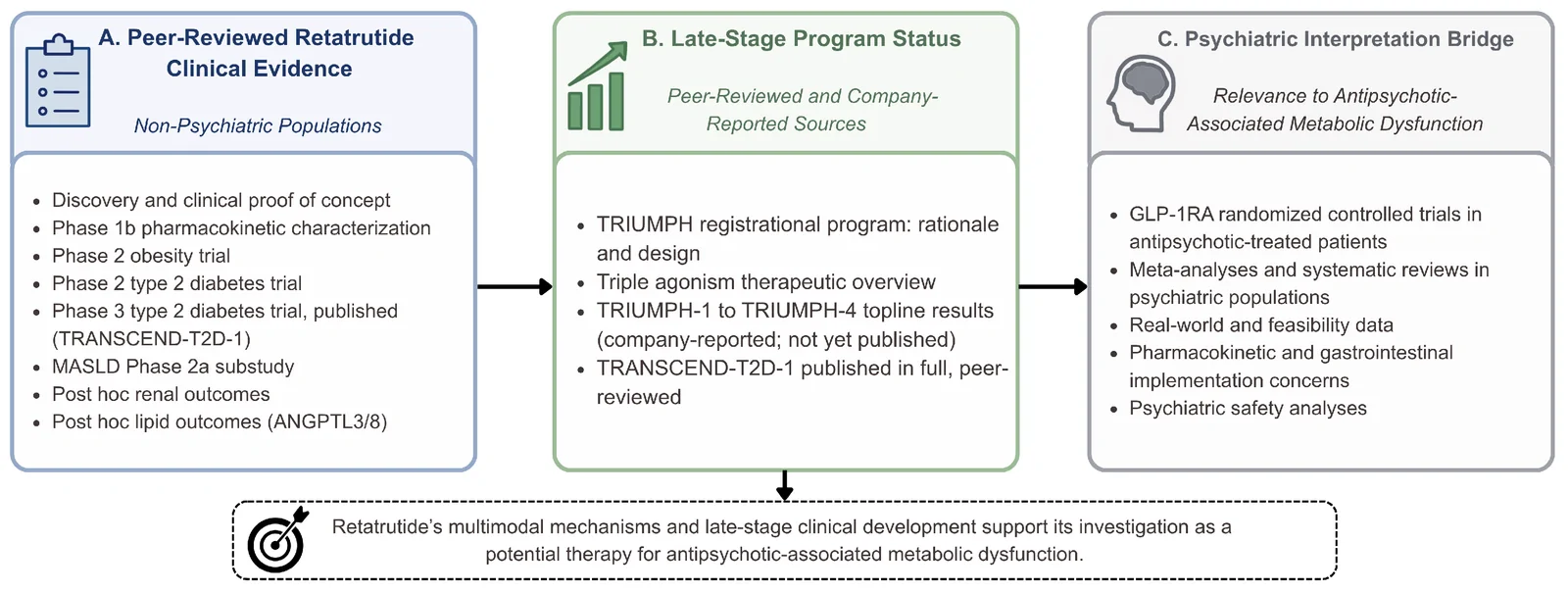
Clinical development of retatrutide and its potential relevance to antipsychotic-associated metabolic dysfunction. (**A**) summarizes peer-reviewed retatrutide clinical data from non-psychiatric populations: the initial discovery and clinical proof-of-concept report [25], Phase 1b pharmacokinetic characterization [90], the Phase 2 obesity [27] and type 2 diabetes [26] trials, the published Phase 3 type 2 diabetes trial [91], a metabolic dysfunction-associated steatotic liver disease substudy [92], and post hoc metabolic analyses of renal [93] and lipid [85] outcomes. (**B**) summarizes the current late-stage evidence base: the registrational program rationale and design [94] and a triple-agonism therapeutic overview [89], together with company-reported topline results from TRIUMPH-1 to TRIUMPH-4 that remain pending peer-reviewed publication [28,29,30], and TRANSCEND-T2D-1, which has been published in full [91]. (**C**) summarizes the indirect evidence relevant to translation: GLP-1 receptor agonist randomized controlled trials in antipsychotic-treated patients [14,15,16,17,18,19,95], meta-analyses and systematic reviews in psychiatric populations [21,22,23,96,97,98], real-world and feasibility data [24,99], pharmacokinetic and gastrointestinal implementation concerns [100,101,102,103,104,105], and psychiatric safety analyses [31,106,107,108]. The summary statement beneath the panels reflects the interpretation advanced in this review, namely that retatrutide’s pharmacological profile and late-stage development provide a rationale for investigation in this population; it is not a claim of demonstrated efficacy, and no trial of retatrutide has been conducted in psychiatric or antipsychotic-treated patients.

Figure 2 summarizes the direct and indirect evidence relevant to evaluating retatrutide in antipsychotic-treated populations, including peer-reviewed retatrutide data from non-psychiatric populations, current late-stage program status, and clinical evidence for GLP-1RAs in psychiatric populations.

Larsen et al. conducted a foundational RCT in 103 adults with schizophrenia spectrum disorders receiving clozapine or olanzapine, who were randomized to liraglutide 1.8 mg/day or placebo for 16 weeks [17]. Liraglutide produced a 5.3 kg greater reduction in body weight than placebo, accompanied by reductions in waist circumference, systolic blood pressure, visceral fat, and LDL-cholesterol. Overall, 63.8% of liraglutide-treated participants achieved normal glucose tolerance compared with 16.0% of those receiving placebo (number needed to treat = 2). No worsening of psychiatric symptoms was reported, and serious adverse events were no more frequent in the liraglutide group. Nausea was more frequent with liraglutide (62% vs. 32%), consistent with the GLP-1RA class profile.

Multiple meta-analyses provide pooled estimates of GLP-1RA effects in this antipsychotic-treated population. Khaity et al. [23] reviewed seven RCTs involving 398 antipsychotic-treated patients; the body-weight meta-analysis pooled five of these and reported a mean reduction of 4.7 kg (95% CI 4.48 to 4.91) vs. placebo, although statistical heterogeneity was substantial (I^2^ = 96%). A sensitivity analysis excluding one outlying trial eliminated the observed heterogeneity (I^2^ = 0%) and yielded a similar estimate of 5.19 kg (95% CI 4.95 to 5.43) [23]. In an individual-participant meta-analysis of three randomized trials comprising CODEX [20], the TAO trial [18,19] and the liraglutide trial of Larsen et al. [17] (*n* = 164), Siskind et al. reported a 3.71 kg greater weight reduction with GLP-1RA than with control (95% CI 2.44 to 4.99), rising to 4.70 kg (95% CI 3.13 to 6.27) in the clozapine/olanzapine subgroup, against 1.5 kg (95% CI −1.47 to 4.47) in patients taking other antipsychotics [22]. Müller Alves et al. [21] extended this to a systematic review of 10 RCTs (*n* = 543). The weight meta-analysis pooled nine of these, one having been excluded for using an active rather than placebo comparator, and found a reduction of 5.03 kg (95% CI 4.01 to 6.04) with no heterogeneity (I^2^ = 0%) [21]. More recently, Stogios et al. evaluated 39 pharmacological interventions across 95 trials in a network meta-analysis [96]. Within that analysis, semaglutide had the largest estimated effect on body weight (−10.98 kg), while liraglutide and exenatide were also among the five most effective interventions, alongside topiramate and metformin; semaglutide and metformin showed clinically meaningful effects of at least 5% weight reduction. Finally, a systematic review of 14 studies involving 139 clozapine- or olanzapine-treated patients reported generally favorable weight and metabolic outcomes [97]. Where psychiatric symptom outcomes were assessed, no worsening of psychotic symptoms was reported. However, psychiatric outcomes were not uniformly prespecified or assessed across the included studies, and the network meta-analysis included interventions other than incretin therapies. These findings therefore represent an absence of reported psychiatric worsening rather than evidence establishing psychiatric safety.

### 6.1. The HISTORI Trial (Semaglutide in Schizophrenia)

Ganeshalingam et al. conducted the HISTORI trial, a randomized, double-blind, placebo-controlled study of semaglutide in 154 antipsychotic-treated patients with schizophrenia spectrum disorders, prediabetes, and obesity [14]. Over 30 weeks, semaglutide produced a 9.21 kg greater reduction in body weight than placebo, with improvements in HbA1c, HDL cholesterol, and triglycerides. Normoglycemia was achieved in 81% of semaglutide-treated participants compared with 19% of those receiving placebo, and scores on the six-item Positive and Negative Syndrome Scale (PANSS-6) did not worsen. HISTORI therefore provides direct evidence that semaglutide can improve metabolic outcomes in antipsychotic-treated patients with schizophrenia without evidence of psychotic symptom worsening over 30 weeks.

### 6.2. The COaST Trial (Semaglutide in Clozapine-Treated Schizophrenia)

Siskind et al. evaluated semaglutide in 31 clozapine-treated patients with schizophrenia spectrum disorders and obesity [15]. This Phase 2 RCT was terminated early because of product non-availability. Over 36 weeks, mean body-weight change was −13.88% with semaglutide vs. −0.42% with placebo, corresponding to a between-group difference of −13.46 percentage points (*p* < 0.0001). Despite the small sample and premature termination, the findings provide preliminary direct evidence that clinically meaningful weight reduction with a GLP-1RA is achievable in clozapine-treated patients, a group with particularly high metabolic risk.

### 6.3. Semaglutide in Early-Stage Metabolic Dysregulation

Sass et al. evaluated semaglutide 1 mg weekly in a multicenter, double-blind RCT involving 73 clozapine- or olanzapine-treated adults with schizophrenia spectrum disorders [95]. Participants had HbA1c values ranging from 5.4 to 7.4% and were not receiving antidiabetic therapy. Over 26 weeks, semaglutide produced a 9.2 kg greater reduction in body weight than placebo (95% CI 5.1 to 13.3; *p* < 0.001). Low-risk HbA1c was achieved by 43% of semaglutide-treated participants compared with 3% of those receiving placebo, with reductions in waist circumference (7.0 cm) and fat mass (6.1 kg). Psychiatric symptoms and psychiatric adverse events did not differ between groups, and gastrointestinal adverse events were common but mild and transient. Suicidality was a prespecified safety endpoint. The two discontinuations for suicidal behavior and all three discontinuations for worsening psychosis occurred in the placebo group; one sudden death and one case of heart failure occurred in the semaglutide group. Participants with baseline suicidal behavior were excluded, which limits inference about suicidality risk. Taken together with HISTORI and COaST, these findings indicate that GLP-1RA therapy can improve weight and selected glycemic outcomes in antipsychotic-treated patients across differing degrees of baseline metabolic impairment.

### 6.4. Exenatide for Olanzapine-Related Weight Gain

Patino et al. conducted a double-blind, placebo-controlled trial of exenatide in 54 olanzapine-treated patients with mood or psychotic disorders [16]. Over 16 weeks, exenatide-treated participants lost 0.5 kg while placebo participants gained 2.6 kg (both *p* < 0.01), with a model-estimated between-group difference of 3.6 kg (*p* = 0.002). Retention was limited: 14 of 24 exenatide-treated participants (58%) completed 16 weeks compared with 20 of 30 (67%) on placebo, and four participants discontinued exenatide because of adverse events or a laboratory safety criterion. These findings highlight tolerability and retention as relevant considerations for future incretin trials in this population.

The broader exenatide evidence includes the TAO trial, a three-month randomized study of 45 participants, of whom 40 completed follow-up [19]. No significant between-group difference in weight loss was observed (2.24 vs. 2.23 kg), although central systolic blood pressure and pulse wave velocity improved with exenatide. Its cognitive substudy found no benefit of exenatide on the Brief Assessment of Cognition in Schizophrenia (BACS) cognitive composite (group interaction *p* = 0.77) or on PANSS ratings [18]. This study provides one of the few direct clinical assessments of cognitive outcomes with GLP-1RA therapy in schizophrenia, but it did not demonstrate a cognitive benefit of exenatide over the three-month study period. Table 3 summarizes the design, population, metabolic entry criteria, intervention and principal metabolic outcome of the randomized and feasibility studies of incretin-based therapy in antipsychotic-treated populations considered in this review.

Direct clozapine-specific randomized evidence for exenatide comes from the CODEX trial, an open-label, parallel-group pilot in which 28 adults with schizophrenia or schizoaffective disorder receiving oral clozapine were randomized 1:1 to once-weekly subcutaneous exenatide 2 mg or usual care for 24 weeks [20]. Six of 14 participants assigned to exenatide lost more than 5% of baseline weight, against one of 14 receiving usual care (*p* = 0.029), and the between-group difference in weight change at 24 weeks was 4.16 kg (*p* = 0.015), accompanied by greater reductions in body mass index, fasting glucose and glycated hemoglobin. Brief Psychiatric Rating Scale scores did not differ between the groups (*p* = 0.808). The trial was open-label and was not powered for efficacy, and fewer than half of the participants felt confident to self-administer injections, so its findings are best read as evidence of feasibility and tolerability in a clozapine-treated population rather than as a definitive efficacy estimate.

### 6.5. Real-World Incretin Evidence and Remaining Evidence Gaps

Varghese et al. analyzed real-world weight outcomes associated with incretin-based therapy across SGA categories in 66,574 adults [24]. Weight reduction was greater among patients receiving metabolically lower-risk SGAs (−3.02 kg, 95% CI −3.29 to −2.74) than among those receiving clozapine or olanzapine (−1.33 kg, 95% CI −2.04 to −0.62), although the absolute difference was modest. In both groups, the reduction achieved remained well below the magnitude of antipsychotic-induced weight gain. Whether the apparent difference reflects antipsychotic-specific pharmacodynamic effects, differences in patient characteristics, or residual confounding cannot be determined from these observational data. If the attenuated response among patients receiving high-risk SGAs is real, it would provide a rationale for evaluating agents with greater demonstrated weight-loss efficacy in obesity trials, since this is the subgroup carrying the greatest metabolic burden. Whether retatrutide’s added glucagon-mediated basal energy expenditure would produce clinically meaningful weight reduction in clozapine- or olanzapine-treated patients requires prospective evaluation. A corresponding evidence gap is present in the randomized trial literature. The most comprehensive network meta-analysis of pharmacological interventions for AIWG to date included 95 randomized trials and 39 interventions but no dual ortriple receptor incretin agonists, because no randomized trials of these agents have been conducted in this population [96]. The authors identified newer incretin therapies, including tirzepatide, as priorities for dedicated study in schizophrenia. The same evidence gap applies to retatrutide; its potential role remains hypothesis-generating rather than evidence of efficacy in antipsychotic-treated patients.

These findings from Varghese et al. [24] require cautious interpretation. The analysis used electronic health records rather than randomized trial data [24]. Although exposed patients were propensity-matched to unexposed patients in a one-to-four ratio, and recent users of other weight-loss medications or metformin in the preceding three months were excluded, residual confounding by indication remains possible. Antipsychotic dose and duration of exposure were not accounted for. The interval around the high-risk estimate is also wide, spanning −2.04 to −0.62 kg. The observed difference between SGA categories therefore requires prospective confirmation, and its mechanism cannot be determined from these retrospective data.

Liu et al. compared 1809 tirzepatide users who had schizophrenia spectrum disorders with 1809 propensity-matched non-users, 3618 patients in total [106]. Over one year, tirzepatide exposure was associated with lower rates of all-cause mortality (HR 0.18, 95% CI 0.06 to 0.51), hospitalization (HR 0.75, 95% CI 0.61 to 0.92), and suicidal behavior (HR 0.43, 95% CI 0.27 to 0.70). None of these outcomes is a weight endpoint, which raises the possibility that incretin-based therapies act through mechanisms beyond weight loss. These observational associations generate hypotheses but do not establish that tirzepatide caused the observed risk reductions.

These findings require caution given the retrospective design [106]. The magnitude of the observational mortality association (HR 0.18) is unusually large for a one-year observational estimate, far exceeds effects reported in any cardiovascular outcome trial to date, and should not be interpreted causally. Residual confounding, including channeling bias not eliminated by propensity matching, may have contributed to the association, and the one-year follow-up limits inference regarding sustained mortality effects. These findings should therefore be regarded as hypothesis-generating rather than definitive.

A feasibility study by Heald et al. provides real-world inpatient evidence [99]. Fifteen adults with schizophrenia or schizoaffective disorder and a BMI of at least 30, treated in a secure unit, received semaglutide for 2 to 6 months. Individual weight change ranged from a 1% increase to a 12% reduction, with a median reduction of 5%. HbA1c normalized in all participants with baseline non-diabetic hyperglycemia, and quality-of-life measures improved. Six of the fifteen participants discontinued before study completion: four because of medical concerns and two after discharge, when funding was no longer available to continue treatment. These findings illustrate both the feasibility of incretin treatment and the practical implementation barriers relevant to future trials in similar psychiatric settings.

## 7. Incretin-Based Anti-Obesity Pharmacotherapy in Antipsychotic-Treated Populations: Evidence Across Agents

Table 4 summarizes the available evidence for anti-obesity pharmacotherapies in antipsychotic-treated populations and distinguishes these agents from those for which relevance remains theoretical.

The evidence base summarized in Table 4 is expanding but remains incomplete [21,98]. Among the agents summarized here, semaglutide has been evaluated in multiple dedicated RCTs in antipsychotic-treated populations, whereas tirzepatide evidence is currently limited to observational and case-level data [106,111]. Retatrutide has produced substantial weight reduction in non-psychiatric obesity trials but has not been evaluated in a psychiatric or antipsychotic-treated population. This absence of direct evidence represents a priority gap for future research.

## 8. Retatrutide Late-Stage Development and Evidence-Quality Considerations

### 8.1. Program Overview

Retatrutide’s late-stage clinical development includes the TRIUMPH Phase 3 program, which evaluates retatrutide in obesity and obesity-related complications, as well as the separate TRANSCEND-T2D program in type 2 diabetes [89,94]. The TRIUMPH program includes TRIUMPH-1 through TRIUMPH-9 and TRIUMPH-Outcomes [112].

Company-reported topline results from TRIUMPH-4, which enrolled adults with obesity or overweight and knee osteoarthritis, were released in December 2025 and described dose-dependent weight reductions of up to 28.7% at 68 weeks [28]. Company-reported topline results from TRIUMPH-2 and TRIUMPH-3 followed in July 2026 [30]. TRIUMPH-2 randomized 1152 adults with type 2 diabetes and obesity or overweight to retatrutide 4 mg, 9 mg, 12 mg, or placebo for 80 weeks; efficacy-estimand weight reductions were 12.7%, 19.1%, and 20.8%, respectively, against 4.0% with placebo, with HbA1c reductions of 1.4 to 1.6 percentage points from a mean baseline of 7.7%. TRIUMPH-3 randomized 1949 adults with severe obesity and established cardiovascular disease, with or without type 2 diabetes, to 9 mg, 12 mg, or placebo, with weight reductions of 21.6% and 22.6% against 3.2% with placebo. Major adverse cardiovascular events (MACE) occurred less frequently than anticipated in both arms, and neither prespecified comparison excluded the null: the hazard ratio was 0.82 (95% CI 0.55 to 1.22) for five-component MACE and 1.12 (95% CI 0.64 to 1.96) for three-component MACE. These findings are company-reported and have not undergone peer review.

Company-reported topline results from the TRIUMPH-1 obesity trial (*n* = 2339) described dose-dependent weight reductions of up to 28.3% at 80 weeks [29]. At the highest dose, 45.3% of participants were reported to have achieved at least 30% weight reduction. The company report also described concurrent improvements in non-HDL cholesterol, triglycerides, and systolic blood pressure [29]. A prespecified blinded extension to 104 weeks enrolled 532 participants with a baseline BMI ≥ 35 who had completed 80 weeks without discontinuing treatment. Participants were escalated to a maximum tolerated dose of 9 mg or 12 mg; among those originally assigned to 12 mg, the reported mean weight reduction reached 30.3%, measured from the extension cohort’s own baseline of 121.7 kg rather than the main-trial baseline of 112.7 kg. Because enrollment eligibility for the extension required completion of 80 weeks without treatment discontinuation, these extension findings should not be generalized to the full randomized population.

TRANSCEND-T2D-1 was a 40-week Phase 3 trial involving 537 adults with type 2 diabetes inadequately controlled by diet and exercise alone, with a mean disease duration of 2.5 years [91]. From a mean baseline HbA1c of 7.9%, HbA1c fell by 1.7% to 1.9% with retatrutide compared with 0.8% with placebo, and mean body-weight reductions ranged from 11.5% to 15.3% compared with 2.6% with placebo. Estimated treatment differences for HbA1c ranged from −0.88 to −1.12 percentage points vs. placebo (all *p* < 0.0001); these results were reported using the treatment-regimen estimand [91].

### 8.2. Efficacy Data and Evidence-Quality Considerations

The publication status of the available Phase 3 evidence differs across trials. TRANSCEND-T2D-1 has been published in full, peer-reviewed form [91], whereas the TRIUMPH-1 and TRIUMPH-4 results discussed here remain company-reported topline findings that have not undergone full peer-reviewed publication [28,29]. The TRIUMPH-2 and TRIUMPH-3 topline results are of the same evidence class, and their detailed results are stated to be pending presentation and peer-reviewed publication [30]. Accordingly, all four TRIUMPH findings should be interpreted as preliminary. The TRIUMPH-4 weight figures cited here are efficacy-estimand results, evaluating treatment effect prior to discontinuation of study drug or another intercurrent event. The corresponding treatment-regimen estimand, which incorporates outcomes irrespective of treatment adherence, yielded mean weight reductions of 20.0% for 9 mg and 23.7% for 12 mg, compared with 4.6% with placebo [28]. Mean baseline weight in TRIUMPH-4 was 112.7 kg (BMI 40.4 kg/m^2^). Secondary outcomes in the TRIUMPH-4 release were in some cases post hoc and were not controlled for multiplicity; systolic blood pressure, for example, was disclosed for the 12 mg arm only (−14.0 mmHg) [28]. The principal TRIUMPH-1 figures are likewise derived from the efficacy estimand. Under the treatment-regimen estimand, mean weight reductions were 23.7% with 9 mg and 25.0% with 12 mg, compared with 3.9% with placebo; mean baseline body weight was 112.7 kg and mean BMI was 40.0 kg/m^2^ [29]. TRANSCEND-T2D-1 reported treatment-regimen estimand results, whereas the Phase 2 obesity trial reported its primary results on the efficacy estimand, with a hybrid estimand used in supplementary analyses. Because these estimands address different assumptions regarding treatment discontinuation and other intercurrent events, numerical results should not be compared across trials without accounting for the estimand used. The published Phase 3 trial is therefore reported on the more conservative of the two principal estimands and the topline trials on the less conservative one, which readers should weigh when comparing figures across them.

Within TRIUMPH-4, the 12 mg efficacy-estimand analysis reported a mean body-weight reduction of 28.7% at 68 weeks, with 58.6% of participants achieving at least 25% weight reduction [28]. For descriptive context, weight-loss estimates from the pivotal semaglutide, tirzepatide, and retatrutide obesity trials are summarized separately in Table 2 [12,13,27,28]. These findings indicate substantial weight reduction within that trial; they should not be interpreted as demonstrating superiority over semaglutide, tirzepatide, or other anti-obesity therapies in the absence of head-to-head comparative evidence.

The magnitude of antipsychotic-associated weight gain underscores the need for effective metabolic interventions. A network meta-analysis of 137 randomized trials involving 35,007 participants, with a median follow-up of 45 weeks, placed clozapine second only to chlorpromazine for weight gain, with a mean difference to placebo of 4.21 kg (95% CrI 3.03 to 5.42) [113]. In a separate eight-year follow-up, patients maintained on clozapine gained an average of 11.7 kg [67]. These data illustrate the long-term metabolic burden of clozapine treatment but do not establish that the magnitude of weight reduction observed with retatrutide in general-obesity trials would be reproduced in this population. That question requires direct study in clozapine-treated patients.

Post hoc Phase 2 analyses have also described renal outcomes in non-psychiatric populations. Heerspink et al. reported a 31.5% reduction in urine albumin-to-creatinine ratio and an increase in eGFR of 8.48 mL/min per 1.73 m^2^ with retatrutide 12 mg in participants with obesity [93]. Whether similar renal effects occur in antipsychotic-treated populations is unknown. Similarly, a Phase 2a substudy by Sanyal et al. in participants with metabolic dysfunction-associated steatotic liver disease reported dose-dependent reductions in hepatic fat with retatrutide, including reductions of up to 82.4% and hepatic fat levels below 5% in 27% to 86% of participants across dose groups [92]. These findings provide additional non-psychiatric metabolic evidence but have not been evaluated in antipsychotic-treated patients.

A safety implication follows from these data. Glucagon receptor agonism is counter-regulatory and could in principle worsen glycemia, which would matter in a population with elevated rates of new-onset diabetes. The available evidence points the other way: a peer-reviewed Phase 2 trial in type 2 diabetes produced dose-dependent HbA1c reductions of up to approximately 2.0% [26], and TRANSCEND-T2D-1 reported reductions across doses with no severe hypoglycemia [91]. These data indicate that the GLP-1 and GIP components outweigh glucagon-mediated hepatic glucose output at the population level. Whether that net effect holds in clozapine- or olanzapine-treated patients with baseline glucometabolic dysfunction is untested and warrants dedicated glycemic monitoring.

## 9. Safety Considerations and Potential Pharmacokinetic Interactions

### 9.1. Dysesthesia and Its Differentiation from Antipsychotic-Associated Neurological Effects

TRIUMPH-4 identified a dose-dependent dysesthesia signal, with reported rates of 8.8% at 9 mg and 20.9% at 12 mg, against 0.7% with placebo [28]. Because TRIUMPH-4 did not enroll an antipsychotic-treated psychiatric population, the relevance of this signal to such patients remains uncertain. Dose-related dysesthesia was also reported in TRIUMPH-1, at rates of up to 12.5% vs. 0.9% with placebo [29]. In TRANSCEND-T2D-1, the only peer-reviewed trial of the five, dysesthesia occurred in 4%, 2%, and 4% of participants receiving 4 mg, 9 mg, and 12 mg, respectively, and in no placebo participants, so rates did not rise with dose in that trial [91]. In TRIUMPH-2, dysesthesia occurred in 4.5%, 5.6%, and 7.3% of participants receiving 4 mg, 9 mg, and 12 mg against 0.7% with placebo, and in TRIUMPH-3 in 6.4% at both 9 mg and 12 mg against 1.3% [30]. Rates rose with dose in TRIUMPH-1, TRIUMPH-2, and TRIUMPH-4, but not in TRIUMPH-3 or TRANSCEND-T2D-1. The rate reported in TRIUMPH-4 is therefore the highest across the five Phase 3 trials and may reflect its osteoarthritis population. In TRIUMPH-1, these events were generally mild to moderate, most resolved during treatment, and most affected participants continued retatrutide [29]; in TRIUMPH-4, events were generally mild and rarely led to discontinuation [28]. In TRANSCEND-T2D-1, most events were likewise mild to moderate and resolved during treatment, although three participants reported severe dysesthesia and two of these discontinued study drug [91]. Ahern et al. published the first case report of tirzepatide-associated allodynia and dysesthesia, and similar reports exist for semaglutide [114]; such sensory events may therefore not be unique to retatrutide, although case-level evidence is insufficient to establish a class effect.

The mechanism underlying retatrutide-associated dysesthesia remains unknown. Hypotheses involving glucagon- or GIP-related sensory signaling, and physiological changes accompanying rapid weight loss, have been proposed, but none has been established in humans [115,116,117]. These mechanisms should therefore be regarded as speculative rather than explanatory.

In antipsychotic-treated patients, attribution may be complicated by pre-existing sensory symptoms: many antipsychotics, notably first-generation agents and, among SGAs, risperidone, are associated with extrapyramidal and peripheral neurological effects [118]. Distinguishing a retatrutide-associated sensory event from pre-existing antipsychotic-related phenomena would therefore require baseline characterization before exposure and prospective reassessment during treatment; specific proposed instruments and timing are set out in Section 10.3.

### 9.2. Potential Pharmacokinetic Interactions with Antipsychotics

Clozapine depends substantially on CYP1A2 metabolism, whereas olanzapine is cleared through multiple pathways that include CYP1A2 and UGT1A4 [62,100,119]. Both drugs show clinically relevant pharmacokinetic variability, making potential changes in exposure an important consideration for future retatrutide studies. As a peptide, retatrutide would not be expected to inhibit or induce CYP enzymes directly, although this has not been formally evaluated; any effect on antipsychotic exposure would therefore be expected to occur indirectly. Two indirect mechanisms are plausible: substantial changes in body weight could alter antipsychotic exposure at a stable dose, and delayed gastric emptying could alter the absorption kinetics of orally administered antipsychotics. Neither has been studied with retatrutide in antipsychotic-treated patients, and the direction and magnitude of any change are unknown. This framework therefore identifies potential interactions requiring study; it does not predict that retatrutide will increase or decrease clozapine or olanzapine concentrations. Given this uncertainty, dedicated early-phase pharmacokinetic interaction studies should precede an efficacy trial in this population, as discussed in Section 10.2.

### 9.3. Clozapine: CYP1A2 Predominance and Clinical Vulnerability

Clozapine is metabolized primarily by CYP1A2, with additional contributions from CYP2C19, CYP2D6, and CYP3A4 [100], conferring vulnerability to interactions with tobacco smoking, which induces CYP1A2 and is highly prevalent in schizophrenia [119,120], and with fluvoxamine, a potent CYP1A2 inhibitor [100]. Phase 1b retatrutide data from Urva et al. demonstrated dose-proportional pharmacokinetics and a terminal half-life of approximately six days, supporting once-weekly administration [90]; however, dedicated interaction studies with antipsychotics have not been performed.

A case report provides indirect clinical evidence that substantial weight change may coincide with clinically relevant changes in clozapine exposure. Approximately 4 kg of weight loss through lifestyle modification in a clozapine-treated patient was associated with a 47% reduction in clozapine plasma levels (from 736 to 387 ng/mL) despite stable dosing [101]. This single-patient observation cannot establish causality or predict the direction or magnitude of any concentration change during retatrutide treatment.

A separate theoretical interaction concerns delayed gastric emptying [102], which could alter the rate and extent of oral clozapine absorption. This effect may be particularly relevant given that clozapine exhibits large interindividual pharmacokinetic variability, which persists even after accounting for identified covariates such as age, sex, smoking status, and CYP1A2 activity [119]. Given this baseline variability, the net pharmacokinetic effect cannot be predicted and should be measured directly in dedicated interaction studies.

### 9.4. Olanzapine: Multienzyme Metabolism and Pharmacokinetic Variability

Olanzapine undergoes hepatic oxidation and conjugation: its principal metabolite is the 10-N-glucuronide, formed largely by UGT1A4, while desmethyl-olanzapine and 2-hydroxymethyl-olanzapine are generated by CYP1A2 and CYP2D6 [62,100]. Pharmacogenetic variation alone does not appear to explain this pharmacokinetic variability. Zubiaur et al. report the Dutch Pharmacogenetics Working Group’s conclusion that CYP2D6 poor- and intermediate-metabolizer phenotypes have no relevant impact on olanzapine’s clinical effects, and judge CYP1A2, CYP2D6, and UGT1A polymorphisms to be unrelated, or related only weakly, to variability in olanzapine response [62]. Consistent with this, Fekete et al. found CYP1A2 expression, rather than CYP1A2 genotype, to be the primary determinant of interindividual variation in olanzapine concentrations [103]. For detecting within-person changes in olanzapine exposure during a future interaction study, serial plasma-concentration measurements would therefore be more directly informative than genotype alone.

Whether retatrutide-associated gastric-emptying delay or substantial weight change alters olanzapine exposure has not been studied. The direction and magnitude of any change cannot be predicted from olanzapine’s metabolic pathway distribution, and future pharmacokinetic studies should therefore measure serial olanzapine concentrations rather than assume a predictable increase or decrease. Current pharmacogenetic evidence does not provide a basis for identifying CYP2D6 poor metabolizers as a uniquely vulnerable subgroup in this context.

### 9.5. Clozapine-Induced Gastrointestinal Hypomotility and Potential Additive Dysmotility

Clozapine-induced gastrointestinal hypomotility (CIGH) is an under-recognized but potentially life-threatening adverse effect; recent UK pharmacovigilance data from Flanagan et al. highlight its clinical burden [104]. CIGH is related in part to clozapine’s antimuscarinic effects, notably potent muscarinic M3 receptor antagonism, and can manifest as constipation, ileus, and, in severe cases, bowel obstruction.

The intersection of CIGH with GLP-1RA-induced gastric-emptying delay creates a theoretical risk of compounded dysmotility. Jalleh et al. described consequences of delayed gastric emptying with GLP-1RAs and tirzepatide, including retained gastric contents at endoscopy, aspiration risk, and altered drug absorption [105]. In clozapine-treated patients already predisposed to gastrointestinal hypomotility, additional slowing related to retatrutide’s GLP-1 activity represents a clinically important theoretical concern. No study has quantified whether combined exposure produces additive dysmotility. Future trials should therefore prespecify active gastrointestinal surveillance and stopping criteria; specific proposed trial-monitoring elements are discussed in Section 10.3.

Collectively, dysesthesia, potential pharmacokinetic interactions, and gastrointestinal hypomotility represent prespecified safety domains that should be evaluated prospectively in any future retatrutide trial involving clozapine- or olanzapine-treated patients.

### 9.6. Psychiatric Safety and Pharmacovigilance Considerations

A meta-analysis by Pierret et al. of 80 randomized placebo-controlled trials involving 107,860 participants with overweight, obesity, or diabetes reported no significant difference in serious or non-serious psychiatric adverse events with GLP-1RA treatment, alongside improvements in restrained and emotional eating and in quality of life [107]. These data derive predominantly from non-psychiatric metabolic populations and cannot establish retatrutide-specific psychiatric safety in schizophrenia spectrum disorders. A systematic review by Sa et al. of 38 studies found no evidence of psychotic symptom exacerbation in the available schizophrenia literature, with signals of benefit in depression, binge eating, and alcohol use [31]. However, the review also identified a pharmacovigilance signal for suicidal ideation in specific co-prescribing contexts, with semaglutide alongside antidepressants or benzodiazepines (reporting odds ratios 4.45 and 4.07, respectively). A dedicated meta-analysis by Ebrahimi et al. of 27 RCTs (32,357 participants receiving a GLP-1RA and 27,046 receiving placebo) found no significant difference in suicide or self-harm rates, which were very low in both groups at 0.044 and 0.040 events per 100 person-years (rate ratio 0.76, 95% CI 0.48 to 1.21; *p* = 0.24) [108]. The discrepancy between pharmacovigilance findings and randomized trial data means that the observational signal should not be interpreted as establishing causality. Importantly, these class-level data do not establish the psychiatric safety of retatrutide itself, which has not been studied in a psychiatric population. That absence is structural rather than incidental: the Phase 2 obesity trial reported no psychiatric outcomes [27], and TRANSCEND-T2D-1 excluded severe psychiatric disorder at entry [91]. Future trials should include prespecified assessment of psychotic symptoms and suicidality.

### 9.7. Nutritional and Eating-Behavior Considerations for Future Trials

GLP-1RAs alter appetite and eating behavior, with studies reporting reductions in emotional or binge-eating symptoms in some populations [31,107]. In individuals with pre-existing restrictive eating pathology, however, marked appetite suppression could theoretically worsen inadequate nutritional intake. This concern has not been evaluated with retatrutide in schizophrenia spectrum disorders. Substantial pharmacological weight loss may also include reductions in lean mass; however, lean-mass reduction should not be equated with sarcopenia in the absence of functional impairment, as discussed in Section 5.1. Future trials should prioritize clinically meaningful metabolic outcomes rather than maximal weight reduction and should incorporate baseline and serial assessment of nutritional status and eating-disorder pathology. Trial eligibility criteria should explicitly address active restrictive eating disorders, low body weight, and clinically significant frailty.

Three considerations deserve particular emphasis. First, the prospect of profound, sustained appetite suppression raises a theoretical concern that a triple agonist could entrench restrictive eating patterns in vulnerable individuals. Case-level, observational, and pharmacovigilance literature has raised concern about the worsening or emergence of restrictive eating disorders during GLP-1RA treatment, particularly among individuals with prior disordered eating [121]. These reports do not establish a causal relationship. Observational data have reported new eating-disorder diagnoses among GLP-1RA-treated individuals with pre-existing mental-health conditions at more than twice the rate seen in those without [121], although residual confounding and differences in baseline vulnerability limit causal interpretation. Such findings support careful assessment in psychiatric trials but do not demonstrate increased risk specifically in schizophrenia spectrum disorders. Second, preclinical and translational findings indicate that GLP-1 signaling modulates reward salience, response, and learning, as well as aversion and general cognitive function [122]; the resulting modulation of reward-related eating may further complicate the behavioral effects of these agents and is incompletely understood. The psychiatric-effects literature similarly emphasizes that the neuropsychiatric actions of these agents extend well beyond appetite and glycemic control [31]. Third, and following directly from these risks, screening should extend beyond formally diagnosed eating disorders to subclinical restrictive patterns. This is especially pertinent given the high but under-recognized comorbidity of eating disorders in schizophrenia, where binge-eating and night-eating syndromes occur in approximately 10% of patients and anorexia nervosa in an estimated 1% to 4%, and where the clearest practical recommendation of the comorbidity literature is systematic screening for and assessment of eating pathology [123]. Any future trial or clinical use of retatrutide in this population should therefore incorporate structured eating-behavior and nutritional screening at baseline and throughout treatment, rather than monitoring weight alone.

## 10. A Framework for Future Trial Development

This section sets out how the preceding rationale could be tested, rather than how retatrutide should be used clinically. Retatrutide remains investigational, has not been studied in any psychiatric population, and no direct evidence supports its use in antipsychotic-treated patients. Nothing here constitutes clinical guidance. The eligibility criteria, thresholds, monitoring intervals, and stopping rules proposed below are unvalidated design suggestions; where specific numerical values are proposed, they should be understood as conservative research estimates rather than empirically validated clinical thresholds.

### 10.1. Defining the Trial Population

The population most directly aligned with the rationale developed in this review comprises adults with schizophrenia spectrum disorders receiving clozapine or olanzapine who have established antipsychotic-associated metabolic dysfunction. This population combines high antipsychotic-related metabolic liability, limited feasibility of therapeutic substitution in appropriately selected patients, and the most directly relevant existing GLP-1RA evidence [14,15,17,95].

Existing metabolic care should serve as the background against which a retatrutide trial is designed rather than as a treatment sequence into which retatrutide is inserted. Consensus guidance recommends metabolic assessment before a second-generation antipsychotic is started, with weight reassessed at 4, 8, and 12 weeks and quarterly thereafter, and blood pressure, fasting plasma glucose, and lipids at longer intervals [124]; it also supports continuing dietary and lifestyle interventions alongside pharmacological management rather than sequencing them ahead of it [68]. Metformin has the most established evidence among pharmacological strategies for preventing or treating antipsychotic-associated weight gain. The Cochrane review by Agarwal et al. found low-certainty evidence that metformin may be effective in preventing weight gain (MD −4.03 kg, 95% CI −5.78 to −2.28), while topiramate did not appear effective, with a point estimate of similar magnitude but with a confidence interval crossing zero (MD −4.82 kg, 95% CI −9.99 to 0.35; three studies, 168 participants) [125]. Choi et al. reached a different conclusion, favoring topiramate and aripiprazole among adjunctive agents in olanzapine- or clozapine-treated patients [126], and broader reviews of psychotropic drug-related weight gain reach comparable conclusions about the limited efficacy of currently available options [127]. Among incretin agents, Lee et al. identified liraglutide as the best-evidenced licensed option [128]. A network meta-analysis of pharmacological interventions for antipsychotic-induced weight gain ranked metformin 750 mg combined with lifestyle modification as the single most effective established approach (−7.5 kg, 95% CI −12 to −2.8), ahead of topiramate 200 mg and metformin alone [129]. GLP-1RAs have additionally demonstrated weight and metabolic effects in antipsychotic-treated populations, as reviewed in Section 6 [14,15,17,95].

The limitations of existing interventions do not justify positioning retatrutide at any specific point in a treatment sequence; such an inference would require comparative evidence that does not exist. Persistent unmet metabolic need instead provides a rationale for conducting a dedicated trial, not for proposing a particular ordering of therapies.

### 10.2. Prerequisite Pharmacokinetic Evaluation

Dedicated early-phase pharmacokinetic interaction studies should precede any efficacy trial in antipsychotic-treated patients. Clozapine depends substantially on CYP1A2 metabolism, whereas olanzapine is cleared through multiple pathways that include CYP1A2 and UGT1A4 (Section 9.2). Retatrutide is not expected to produce a direct CYP-mediated interaction, but delayed gastric emptying and substantial changes in body weight or composition could potentially alter antipsychotic absorption kinetics or exposure. Existing evidence does not permit the direction of any weight-related effect on clozapine exposure to be predicted: a systematic review of clozapine population pharmacokinetic models found no consistent effect of body weight in pooled models, while noting conflicting studies associating greater body weight or body mass index with higher clozapine concentrations [119]. Both drugs are also lipophilic and extensively distributed into tissue: olanzapine has a volume of distribution of 16.4 ± 5.1 L/kg [130], and in rats, brain clozapine concentrations exceeded serum concentrations 15.8-fold [131]. Adiposity is itself associated with circulating clozapine; in a reanalysis of a double-blind trial, each 1 kg gained during clozapine treatment was associated with a 1.4% increase in total plasma clozapine concentrations (95% CI 0.55 to 2.3), and each one-point increase in percentage body fat with a 5.4% increase [132]. Substantial and rapid loss of adipose tissue could therefore alter apparent volume of distribution, tissue sequestration, or redistribution, and plasma concentrations may not fully reflect tissue or target-site exposure. This is mechanistically plausible rather than established in this setting and should be measured prospectively rather than assumed. The direction and magnitude of any retatrutide-associated change in clozapine or olanzapine exposure are therefore unknown. This uncertainty supports direct pharmacokinetic characterization before efficacy testing rather than assuming that antipsychotic exposure will remain stable during retatrutide treatment.

### 10.3. Proposed Trial Monitoring Requirements

The following are proposed monitoring components for future trials. None has been validated in this context.

**Antipsychotic therapeutic drug monitoring:** Future pharmacokinetic and efficacy protocols should include serial clozapine or olanzapine plasma concentrations at prespecified time points, including baseline and during retatrutide dose escalation, as well as after clinically substantial weight change or an antipsychotic dose change. For exploratory pharmacokinetic characterization, sampling at approximately 5% increments in body-weight reduction could be considered. The 5% value is a conservative, pragmatic research-sampling interval intended to generate the data required for subsequent pharmacokinetic modeling. It is not derived from pharmacokinetic modeling and should not be interpreted as a validated clinical action threshold. Plasma therapeutic drug monitoring, although clinically useful, may not fully capture changes in tissue distribution.

**Sensory and neurological assessment:** Because pre-existing extrapyramidal or sensory symptoms may complicate attribution of a new sensory adverse event (Section 9.1), future trials should characterize these domains before retatrutide exposure and reassess them prospectively during treatment. Established movement-disorder instruments, such as the Simpson-Angus Scale for antipsychotic-induced parkinsonism and the Barnes Akathisia Rating Scale for akathisia, could characterize competing extrapyramidal phenomena; these assessments should be paired with a structured sensory examination and a validated neuropathic-pain instrument such as the Neuropathic Pain Symptom Inventory, with quantitative sensory testing where feasible. Scheduled reassessment at fixed intervals is preferable to reliance on spontaneous adverse-event reporting, since patients with chronic pre-existing symptoms are less likely to volunteer new ones. Temporal relationship to retatrutide initiation or dose escalation, symptom distribution and quality, reversibility, and blinded adjudication of ambiguous events should be prespecified where feasible.

**Gastrointestinal monitoring:** Future clozapine-retatrutide trials should incorporate structured bowel-habit assessment at baseline and during dose escalation rather than relying solely on spontaneous adverse-event reporting. A prespecified research trigger for clinical review before further escalation could include stool frequency below three bowel movements per week, or new abdominal pain, distension, or vomiting; this threshold has not been validated specifically for retatrutide-treated patients. Suspected ileus or bowel obstruction should constitute a prespecified safety event requiring immediate clinical evaluation and protocol-directed interruption of study treatment. Existing clozapine bowel-management regimens should be documented and managed according to established clozapine-specific guidance [104].

**Body composition and muscle function:** Because incretin-associated weight loss includes a lean-mass component [80,81,82] and baseline sarcopenic risk is elevated in schizophrenia [83], body composition should be measured directly by DXA or bioelectrical impedance rather than inferred from body weight. Muscle function should be assessed alongside mass, since lean-mass reduction identified by imaging does not constitute sarcopenia in the absence of demonstrated functional impairment [84]. Future trials should also prespecify a muscle-preservation strategy. Standardized resistance exercise and an adequate minimum protein intake could be incorporated as protocolized co-interventions [133], while body composition and muscle function should be assessed as prespecified outcomes [79,80,81,82,83,84].

**Psychiatric symptom monitoring:** Future trials should include serial assessment of psychotic symptoms using the PANSS or an equivalent validated measure, together with prespecified monitoring for suicidality; these domains were assessed in previous incretin trials in antipsychotic-treated populations [14,15,95].

**Nutritional and eating-behavior assessment:** Trials should include structured screening for eating-disorder pathology and nutritional status at baseline and at prespecified follow-up visits [121,123]. Protocols should prospectively define criteria for clinical review, dose modification, or study-drug discontinuation in the event of nutritional compromise or a clinically concerning rate or magnitude of weight loss; no retatrutide-specific threshold has yet been validated for this population.

### 10.4. Feasibility Considerations for Trial Design

Several practical factors would condition the design and interpretation of any trial in this population.

Antipsychotic non-adherence affects roughly 40% to 50% of patients, and 48% within the first year of treatment [50,51,52]. A weekly injectable intervention may introduce adherence and treatment-support requirements that differ from those encountered in general-obesity trials. The 40% discontinuation rate reported in the small secure-inpatient feasibility study by Heald et al. [99] supports prespecifying treatment retention and reasons for discontinuation as feasibility outcomes.

Gastrointestinal tolerability may also influence trial retention. Nausea was reported in 62% of liraglutide-treated participants in the Larsen et al. trial [17], while the separate theoretical concern of additive gastrointestinal dysmotility in clozapine-treated patients is discussed in Section 9.5.

Cost and access are further considerations. In the absence of psychiatric indication-specific reimbursement, the cost of triple-agonist therapy may be prohibitive for many patients with severe mental illness, among whom socioeconomic disadvantage is over-represented. Implementation studies should therefore assess treatment access, reimbursement, and continuity after trial completion, because these factors may affect retention and external validity.

Finally, substantial appetite suppression and weight reduction may create nutritional or eating-behavior risks that have not been studied with retatrutide in schizophrenia spectrum disorders. The screening and eligibility measures proposed in Section 9.7 and Section 10.3 are precautionary trial-design proposals rather than responses to an established retatrutide-specific risk in this population.

## 11. Evidence Gaps and Future Research Priorities

The available evidence identifies several domains in which data are derived directly from retatrutide studies outside psychiatric populations, are derived indirectly from other incretin therapies in antipsychotic-treated populations, or remain entirely unknown in the target population. Table 5 summarizes these evidence boundaries and the principal priorities for future research.

1. **Pharmacokinetic interaction studies:** Dedicated early-phase studies should characterize retatrutide-associated changes in clozapine and olanzapine exposure before a Phase 2 efficacy trial is undertaken. The gastric-emptying delay and weight-loss-mediated volume-of-distribution changes described in Section 9 remain theoretical, and neither the direction nor the magnitude of any interaction can currently be predicted.
2. **Absence of psychiatric population data:** No clinical trial has evaluated retatrutide in any antipsychotic-treated population. Following prerequisite pharmacokinetic interaction characterization, a randomized, placebo-controlled efficacy trial in clozapine- and olanzapine-treated patients should be a research priority.
3. **Gastrointestinal safety in clozapine-treated patients:** Whether retatrutide-mediated gastric slowing compounds clozapine-induced gastrointestinal hypomotility has not been studied [104]. Future trials should prospectively evaluate bowel function and treatment discontinuation related to gastrointestinal events using the monitoring and adjudication procedures set out in Section 10.3.
4. **Dysesthesia characterization in neuropsychiatric populations:** The 20.9% dysesthesia rate at the 12 mg dose warrants dedicated assessment in patients who may already have baseline extrapyramidal or sensory symptoms [28]. Whether retatrutide-associated sensory disturbance is distinguishable from antipsychotic-associated sensory phenomena, and what its time course and reversibility are, remain unanswered; the instruments and timing proposed to address this are set out in Section 10.3.
5. **Lean-mass preservation and body-composition outcomes:** Substantial incretin-associated weight reduction can include a lean-mass component [80,81,82], but whether retatrutide’s glucagon receptor activity increases the proportion or absolute magnitude of that loss has not been established. This question is particularly relevant in schizophrenia spectrum disorders, where possible sarcopenia has been reported [83]. The body-composition and muscle-function measures proposed to answer it are set out in Section 10.3.
6. **Long-term metabolic and cardiovascular outcomes:** Long-term retatrutide studies should evaluate the durability of weight and metabolic effects and cardiovascular outcomes in clozapine- and olanzapine-treated schizophrenia spectrum disorders. The TRIUMPH program will generate long-term data on general obesity, including cardiovascular mortality endpoints in TRIUMPH-Outcomes, but general-obesity data cannot establish corresponding benefit in a population with premature cardiovascular mortality and chronic inflammatory burden.
7. **Comparative effectiveness studies:** Head-to-head comparisons of retatrutide, semaglutide, and tirzepatide in antipsychotic-treated populations would be required to determine whether the additional glucagon receptor component provides incremental benefit sufficient to justify the potential for additional adverse effects. Existing cross-trial comparisons cannot answer this question.
8. **Medication adherence outcomes:** Future trials should include antipsychotic adherence or treatment continuity as a prespecified exploratory outcome. Existing literature associates weight gain and obesity with poorer adherence [53,55], but whether pharmacological weight reduction improves antipsychotic adherence has not been established and should be tested directly.
9. **Sex and gender differences in metabolic risk and treatment response:** Sex and gender remain under-explored dimensions of both antipsychotic-associated metabolic dysfunction and incretin response. Early weight-gain meta-analyses, including Allison et al., could not examine sex-specific effects for lack of sex-disaggregated data [64], whereas the Pillinger et al. network meta-analysis identified male sex, alongside higher baseline weight and non-white ethnicity, as a predictor of greater vulnerability to antipsychotic-induced metabolic dysregulation, with the strongest evidence for fasting glucose (*p* = 0.0082) [59]. In the retatrutide Phase 2 obesity trial, sex was a prespecified subgroup and mean percentage weight reduction was greater in women than in men at the 8 mg and 12 mg doses (28.5% and 26.6% vs. 19.8% and 21.9%); the investigators state that whether this reflects sex-dependent differences in body composition, adipose distribution, or hormonal milieu remains to be determined [27]. Whether treatment response differs by sex in antipsychotic-treated patients is unknown; future trials should prespecify sex-stratified analyses and report sex-disaggregated outcomes.

## 12. Limitations

Several limitations of the present review and the underlying evidence base should be acknowledged. First, the Phase 3 evidence differs in publication status. TRIUMPH-1, TRIUMPH-2, TRIUMPH-3, and TRIUMPH-4 results cited throughout this review remain company-reported topline results rather than peer-reviewed publications [28,29,30], whereas TRANSCEND-T2D-1 has been published in full [91]. Accordingly, effect estimates and secondary outcomes reported from these four trials should be regarded as preliminary until complete peer-reviewed reports are available. TRANSCEND-T2D-1 also studied adults whose type 2 diabetes was inadequately controlled by diet and exercise alone, who were insulin-naive, and who had not taken oral or injectable antihyperglycemic medication for at least 90 days before screening; its investigators identify this largely medication-naive population as a limitation that reduces the generalizability of their findings [91]. This differs substantially from the target population considered in this review, in whom ongoing antipsychotic treatment is intrinsic to the clinical question and concomitant pharmacotherapy may be common.

Second, no clinical trial has evaluated retatrutide in a psychiatric or antipsychotic-treated population, and TRANSCEND-T2D-1 excluded severe psychiatric disorder at entry [91]. Consequently, the proposed application to clozapine- and olanzapine-associated metabolic dysfunction remains theoretical and is extrapolated principally from retatrutide studies in obesity and type 2 diabetes together with GLP-1RA evidence in antipsychotic-treated populations.

Third, the proposed pharmacokinetic interactions with clozapine and olanzapine are based on mechanistic reasoning, indirect evidence, and case-level observations rather than dedicated retatrutide-antipsychotic interaction studies. Neither the direction nor the magnitude of any change in antipsychotic exposure can currently be predicted.

Fourth, the reported associations between tirzepatide exposure and mortality, hospitalization, and suicidal behavior in schizophrenia spectrum disorders derive from retrospective observational data and remain vulnerable to residual confounding and channeling bias despite propensity matching [106]. These findings are hypothesis-generating and should not be interpreted causally, as detailed in Section 6.5.

Fifth, the psychiatric incretin literature remains relatively small and heterogeneous, and publication bias or selective reporting may affect the apparent consistency of metabolic and psychiatric outcomes.

Sixth, although a structured literature search was used to inform this narrative review, study selection and synthesis remained author-guided rather than governed by a systematic-review protocol. No de novo quantitative synthesis was performed; effect estimates are therefore reported as given in the original studies and should not be interpreted as directly comparable across heterogeneous populations, interventions, durations, and statistical estimands.

Seventh, body-composition data for retatrutide remain limited, and the magnitude and functional significance of any lean-mass reduction in antipsychotic-treated patients are unknown.

Eighth, efficacy estimates for semaglutide, tirzepatide, and retatrutide originate from separate trials and are presented for descriptive context only, as set out in Section 5.2 and the Table 2 footnote; they cannot establish comparative superiority in the absence of head-to-head trials.

Collectively, these limitations support the need for staged prospective investigation beginning with pharmacokinetic and safety characterization, followed by adequately powered randomized trials with prespecified metabolic, psychiatric, gastrointestinal, neurological, and body-composition outcomes.

## 13. Conclusions

Antipsychotic-associated metabolic dysfunction remains a clinically important and incompletely addressed complication of psychopharmacological treatment. Clozapine and olanzapine retain essential therapeutic roles in schizophrenia spectrum disorders despite their substantial metabolic liability. The combination of substantial metabolic burden in clozapine- and olanzapine-treated patients, emerging GLP-1RA evidence in antipsychotic-treated populations, and retatrutide efficacy in non-psychiatric obesity and type 2 diabetes trials provides a rationale for direct investigation of retatrutide in this setting.

This rationale remains hypothetical rather than an established treatment approach: no retatrutide data exist in any psychiatric or antipsychotic-treated population. The proposed application is therefore based on indirect evidence from retatrutide studies in obesity and type 2 diabetes, together with GLP-1RA evidence in antipsychotic-treated populations and mechanistic extrapolation. Retatrutide should not be regarded as an established therapy for antipsychotic-associated metabolic dysfunction unless efficacy and safety are demonstrated directly in this population.

Retatrutide’s combined GLP-1, GIP, and glucagon receptor agonism engages several pathways relevant to body-weight regulation, glycemic control, and energy metabolism, including glucagon-mediated effects on basal energy expenditure and hepatic metabolism. This combination may correspond more closely to the multifactorial pathology of antipsychotic-associated metabolic dysfunction than single- or dual-receptor incretin therapies, but whether it confers any clinical advantage in antipsychotic-treated patients is unknown and requires direct comparative study. In non-psychiatric obesity populations, the peer-reviewed Phase 2 trial reported mean body-weight reduction of up to 24.2% at 48 weeks [27]. Company-reported Phase 3 topline findings from TRIUMPH-1, TRIUMPH-2, TRIUMPH-3, and TRIUMPH-4 also describe substantial weight reduction [28,29,30], but these results remain pending full peer-reviewed publication and should therefore be interpreted as preliminary. The magnitude of weight reduction observed in general-obesity trials cannot be assumed to occur in antipsychotic-treated patients, and no head-to-head evidence establishes superiority over existing incretin therapies in this population.

Important uncertainties include the absence of retatrutide data in psychiatric populations; dysesthesia observed in retatrutide trials, which was dose-related in most but not all trials; unresolved pharmacokinetic interactions with clozapine and olanzapine; the theoretical possibility of additive gastrointestinal dysmotility with clozapine; and limited information on body-composition and lean-mass effects. Whether any lean-mass reduction translates into clinically meaningful loss of muscle function in schizophrenia spectrum disorders remains unknown.

A staged research program should begin with dedicated pharmacokinetic and safety characterization before proceeding to adequately powered randomized efficacy trials in clozapine- and olanzapine-treated patients. This review therefore provides a trial-development framework for testing retatrutide in antipsychotic-associated metabolic dysfunction. Whether triple receptor agonism ultimately has a clinically meaningful role in this setting can be determined only through direct prospective study.

## Figures and Tables

**Table 1:** Placebo-adjusted weight change and metabolic liability ratings for selected antipsychotics.

Agent	Class	Weight Change vs. Placebo, kg (95% CI) *	Diabetes Liability †	Dyslipidemia Liability †	Overall Metabolic Liability †	Clinical Notes	Refs.
**Clozapine**	SGA	3.01 (1.78 to 4.24)	**High**	**High**	**Very High**	Established treatment of choice for treatment-resistant schizophrenia; long-term weight gain may substantially exceed short-term trial estimates	[10,57,58,59,64,66,67,69]
**Olanzapine**	SGA	2.73 (2.38 to 3.07)	**High**	**High**	**Very High**	Approximately 30% of patients gain more than 7% of baseline body weight during CATIE Phase 1; high overall efficacy is clinically relevant when considering switching strategies	[11,47,59,65]
**Quetiapine**	SGA	1.56 (1.09 to 2.04)	**Moderate**	**Moderate**	**Moderate**	Used across multiple psychiatric indications; intermediate metabolic burden	[47,59,66]
**Risperidone and paliperidone**	SGA	1.28 (0.98 to 1.59)	**Low-Moderate**	**Low**	**Low-Moderate**	Reported as a single node in the source network meta-analysis	[59]
**Asenapine**	SGA	1.17 (0.47 to 1.86)	**Low**	**Low**	**Low**	Relatively low metabolic burden among the agents included in this comparison	[11,59]
**Lurasidone**	SGA	0.48 (−0.01 to 0.97)	**Minimal**	**Minimal**	**Minimal**	Relatively favorable metabolic profile; the confidence interval for weight change includes zero	[11,59]
**Aripiprazole**	SGA	0.34 (−0.16 to 0.84)	**Minimal**	**Minimal**	**Minimal**	Relatively low short-term weight liability; the confidence interval includes zero	[11,59]
**Haloperidol**	FGA	−0.23 (−0.83 to 0.36)	**Minimal**	**Minimal**	**Minimal**	First-generation comparator agent; low short-term weight liability in the source analysis; the confidence interval includes zero	[11,59]
**Ziprasidone**	SGA	−0.28 (−1.15 to 0.59)	**Minimal**	**Minimal**	**Minimal**	Low weight liability in CATIE, and the only CATIE agent with improvement in glycated hemoglobin, total cholesterol and triglycerides; the confidence interval for placebo-adjusted weight change includes zero	[7,47,59]

* Weight change represents the placebo-adjusted mean difference with a 95% confidence interval, taken from the network meta-analysis of Pillinger et al. [59], which pooled 100 randomized trials and 25,952 participants at a median treatment duration of 6 weeks. All weight-change values derive from this single source and estimand, and agents are ordered by descending placebo-adjusted weight change. Risperidone and paliperidone are reported as a single node in the source analysis and are therefore presented together. Confidence intervals that include zero indicate that the weight change was not statistically distinguishable from placebo. These placebo-adjusted short-term differences should not be interpreted as estimates of absolute long-term weight gain; longer-term data are discussed in Section 3.2 and Section 8.2. † Diabetes liability, dyslipidemia liability, and overall metabolic liability are author-derived qualitative classifications, not quantitative effect estimates. They integrate evidence on body-weight change, fasting glucose, incident diabetes, triglycerides, and cholesterol fractions from [7,11,57,58,59,63,64,65,66,68,69]; Refs. [47,48,60,61,62] provide supporting context on CATIE metabolic outcomes and treatment rates, metabolic syndrome prevalence, adjunctive aripiprazole, and olanzapine pharmacogenetics. Liability ratings are color-coded for ease of comparison: blue, minimal; green, low; light green, low-moderate; amber, moderate; red, high; dark red, very high. The color duplicates the rating given in words in each cell and carries no additional information. Agent names and liability ratings are set in bold for navigation only.

**Table 2:** Mechanistic and trial characteristics of incretin-based anti-obesity agents.

Feature	Semaglutide	Tirzepatide	Retatrutide
GLP-1 receptor	Agonist	Agonist	Agonist
GIP receptor	—	Agonist	Agonist
Glucagon receptor	—	—	Agonist
Obesity trial(s) represented	STEP 1 [12]	SURMOUNT-1 [13]	Phase 2 obesity trial [27]; TRIUMPH-1, company-reported topline [29]
Dose evaluated	2.4 mg weekly	15 mg weekly	12 mg weekly (Phase 2 and TRIUMPH-1)
Treatment duration	68 weeks	72 weeks	48 weeks (Phase 2); 80 weeks (TRIUMPH-1)
Study population	Adults with overweight or obesity without diabetes	Adults with obesity, or overweight with ≥1 weight-related complication, without diabetes	Adults with obesity, or overweight with ≥1 weight-related comorbidity, without diabetes
Mean baseline body weight; BMI	105.3 kg; 37.9 kg/m^2^	104.8 kg; 38.0 kg/m^2^	107.7 kg; 37.3 kg/m^2^ (Phase 2); 112.7 kg; 40.0 kg/m^2^ (TRIUMPH-1)
Mean percentage change in body weight from baseline, by estimand	−14.9% (treatment policy); −16.9% (trial product)	−20.9% (treatment regimen); −22.5% (efficacy)	Phase 2: −24.2% (efficacy). TRIUMPH-1: −25.0% (treatment regimen); −28.3% (efficacy); company-reported topline
Appetite and satiety mechanism	GLP-1-mediated satiety; delayed gastric emptying	GLP-1- and GIP-mediated satiety; delayed gastric emptying	GLP-1- and GIP-mediated satiety; delayed gastric emptying
Insulin sensitivity	Improved, largely secondary to weight reduction	Improved; direct GIP receptor agonism may contribute	Improved; direct GIP receptor agonism may contribute
Effect on energy expenditure	Not a primary mechanism	Not a primary mechanism	Glucagon receptor agonism can increase resting energy expenditure; magnitude of retatrutide-specific contribution to weight loss in humans not established
Hepatic lipid handling	Reduced hepatic steatosis with weight loss	Reduced hepatic steatosis with weight loss	Glucagon-mediated hepatic lipid oxidation; reduced ANGPTL3/8 [85]
Evidence in antipsychotic-treated populations	RCTs (HISTORI, COaST, Sass et al.)	Case reports, cohort	None; mechanistic rationale only

Values are drawn from separate trials differing in population, entry criteria, dose, duration, background therapy, missing-data handling, and statistical estimand. They are therefore cross-trial comparisons and do not constitute evidence of comparative superiority; no head-to-head trial of these agents has been conducted. Baseline body weight and body mass index are whole-trial means including placebo groups. Weight-reduction figures are within-group mean changes from baseline rather than placebo-adjusted differences, and each is labeled with the estimand that produced it. Estimand terminology differs between sponsors. The treatment-policy estimand of STEP 1 [12] and the treatment-regimen estimands of SURMOUNT-1 [13] and TRIUMPH-1 [29] all include randomized participants irrespective of adherence; the trial-product estimand of STEP 1 [12] and the efficacy estimands of SURMOUNT-1 [13], the retatrutide Phase 2 trial [27], and TRIUMPH-1 [29] estimate the effect had treatment been continued as assigned. The Phase 2 trial reported no treatment-regimen analysis. TRIUMPH-1 results are company-reported topline data pending peer-reviewed publication. TRIUMPH-2, TRIUMPH-3, and TRIUMPH-4 are not tabulated here because their populations (type 2 diabetes, established cardiovascular disease, and knee osteoarthritis, respectively [28,30]) are not comparable to the general-obesity populations of the other trials; those results are reported in Section 8.1 and Section 8.2. —, no agonist activity at that receptor.

**Table 3:** Characteristics of randomized and feasibility studies of incretin-based therapy in antipsychotic-treated populations.

Study	Design; *n*	Population; Antipsychotic	Metabolic Entry Criteria	Intervention; Duration	Principal Metabolic Outcome	Refs.
Larsen et al. (2017)	RCT; 103	Schizophrenia spectrum; clozapine or olanzapine	Prediabetes with overweight or obesity	Liraglutide 1.8 mg/day; 16 wks	−5.3 kg vs. placebo; 63.8% vs. 16.0% normal glucose tolerance	[17]
Ishøy et al. (2017), TAO	RCT; 45 (40 completed)	Schizophrenia spectrum, non-diabetic; antipsychotic-treated	Obesity	Exenatide weekly; 3 mo	No weight difference (2.24 vs. 2.23 kg); reduced central systolic BP and pulse wave velocity	[18,19]
Ganeshalingam et al. (2025), HISTORI	Double-blind RCT; 154	Schizophrenia spectrum; antipsychotic-treated	Prediabetes with obesity	Semaglutide; 30 wks	−9.21 kg; 81% vs. 19% normoglycemia; improved HbA1c, HDL, triglycerides	[14]
Siskind et al. (2025), COaST	Phase 2 RCT; 31; stopped early for drug supply	Schizophrenia spectrum; clozapine	Obesity	Semaglutide; 36 wks	−13.88% vs. −0.42% body weight (difference −13.46%, *p* < 0.0001); second interim analysis	[15]
Sass et al. (2026)	Multicenter double-blind RCT; 73	Schizophrenia spectrum; clozapine or olanzapine	HbA1c 5.4–7.4%; no antidiabetic therapy	Semaglutide 1 mg weekly; 26 wks	−9.2 kg vs. placebo (95% CI −13.3 to −5.1); 43% vs. 3% low-risk HbA1c	[95]
Patino et al. (2025)	Double-blind RCT; 54	Mood or psychotic disorders; olanzapine	Overweight or obesity	Exenatide; 16 wks	−0.5 kg vs. +2.6 kg (model-estimated difference −3.6 kg, *p* = 0.002); 58% vs. 67% completed 16 weeks	[16]
Siskind et al. (2018), CODEX	Open-label RCT; 28	Schizophrenia or schizoaffective disorder; clozapine	BMI 30–45; with or without stable T2DM	Exenatide 2 mg weekly; 24 wks	6/14 vs. 1/14 achieved >5% weight loss (*p* = 0.029); between-group difference −4.16 kg (*p* = 0.015)	[20]
Heald et al. (2025)	Feasibility; 15; secure inpatient	Schizophrenia or schizoaffective disorder	BMI ≥ 30	Semaglutide; 2–6 mo	Median 5% weight loss; HbA1c normalized in all with baseline hyperglycemia; 40% discontinued	[99]

RCT, randomized controlled trial; BP, blood pressure; wks, weeks; mo, months. All studies evaluated GLP-1 receptor mono-agonists. No randomized trial of a dual ortriple receptor incretin agonist has been conducted in an antipsychotic-treated psychiatric population.

**Table 4:** Evidence for incretin-based anti-obesity pharmacotherapies in antipsychotic-treated populations.

Agent	Mechanism	Mean Weight Loss (General Obesity)	Evidence in Antipsychotic-Treated Populations	Clozapine-Specific Evidence	Psychiatric and Tolerability Findings
Exenatide	GLP-1 receptor agonist	~4.7% (5.1 kg, 24 wks) [109]	RCT in olanzapine-treated patients [16]; TAO RCT in antipsychotic-treated patients with schizophrenia spectrum disorders [18,19]; CODEX RCT in clozapine-treated patients with schizophrenia spectrum disorders [20]	Direct RCT evidence in clozapine-treated patients with schizophrenia (CODEX); open-label pilot, *n* = 28 [20]	No significant change in PANSS ratings or cognitive benefit in the TAO substudy [18]; retention and tolerability limitations were observed in the olanzapine trial [16]; no between-group difference in BPRS in CODEX, with transient nausea, vomiting, dizziness and diarrhea the main adverse events [20]
Liraglutide	GLP-1 receptor agonist	~8.0% (56 wks) [110]	RCT in clozapine- or olanzapine-treated patients with schizophrenia spectrum disorders [17]; systematic-review and meta-analytic support [21,98]	Direct RCT evidence in a mixed clozapine/olanzapine-treated population [17]	No reported worsening of psychiatric symptoms in the cited RCT; nausea frequent (62%) [17]
Semaglutide	GLP-1 receptor agonist	~14.9% (68 wks) [12]	Multiple RCTs in antipsychotic-treated populations: HISTORI [14], COaST [15], and Sass et al. [95]	Direct Phase 2 RCT evidence in clozapine-treated patients with schizophrenia spectrum disorders (COaST) [15]; additional mixed clozapine/olanzapine RCT evidence [95]	No evidence of psychotic symptom worsening in the cited RCTs; suicidality findings were reassuring but are limited by trial design and eligibility criteria [14,15,95]
Tirzepatide	GLP-1/GIP dual receptor agonist	~20.9% (72 wks) [13]	Retrospective propensity-matched cohort of 1809 pairs in schizophrenia spectrum disorders; no randomized psychiatric trial [106]	Case report evidence only [111]	Observational association with lower suicidal behavior (HR 0.43); substantial residual confounding remains possible [106]
Retatrutide	GLP-1/GIP/GCG triple receptor agonist	~24.2% (48 wks) [27]	No data; no clinical trial has evaluated retatrutide in a psychiatric or antipsychotic-treated population	No data	No direct psychiatric safety or tolerability data; relevance to this population remains theoretical

Agents are ordered by increasing weight-loss efficacy in general-obesity trials, an ordering that runs inversely to the strength of evidence available in antipsychotic-treated populations: exenatide, liraglutide, and semaglutide have been studied in randomized controlled trials in this population, tirzepatide only in observational data with high confounding risk, and retatrutide not at all. The retatrutide row therefore represents theoretical potential, as no psychiatric population data exist. Weight-loss figures are within-group changes from the cited general-obesity trials and are not directly comparable, since trial duration, population, and statistical estimand differ; the estimands are specified in Table 2 for semaglutide, tirzepatide, and retatrutide. Data from [12,13,14,15,16,17,18,19,20,21,27,95,98,106,109,110,111].

**Table 5:** Evidence status and key unknowns for retatrutide in antipsychotic-associated metabolic dysfunction.

Domain	Direct Retatrutide Evidence Outside Psychiatric Populations	Relevant Evidence in Antipsychotic-Treated Populations	Unknown in Retatrutide-Treated Psychiatric Populations
Weight reduction	Substantial body-weight reduction demonstrated in adults with overweight or obesity [27,28,29]	GLP-1RAs reduce body weight in clozapine- and/or olanzapine-treated patients [14,15,17,95]	Magnitude, durability, and tolerability of retatrutide-associated weight reduction; no trial conducted
Glycemic outcomes	HbA1c reduction demonstrated in type 2 diabetes [26,91]	GLP-1RAs improve glycemic outcomes in antipsychotic-treated patients [14,17,95]	Effect in antipsychotic-associated dysglycemia specifically
Energy expenditure	Glucagon administration can increase resting energy expenditure in non-psychiatric human studies [78]; retatrutide-specific contribution remains uncertain	No direct evidence addressing this mechanism in antipsychotic-treated patients	Whether this offsets sedation-related reductions in physical-activity energy expenditure; no data address centrally mediated sedation
Psychiatric safety	Psychiatric outcomes were not reported in the Phase 2 obesity trial [27]; TRANSCEND-T2D-1 excluded severe psychiatric disorder at entry [91]	GLP-1RA trials and meta-analyses have not reported psychotic symptom worsening, although psychiatric outcomes were not uniformly designed to establish safety [14,15,17,21,22,23]	Retatrutide-specific psychiatric safety; no data in any psychiatric population
Pharmacokinetic interactions	Retatrutide delays gastric emptying [102]; no dedicated interaction study with clozapine or olanzapine	Clozapine depends on CYP1A2 metabolism; olanzapine has multienzyme metabolism including CYP1A2 [62,100,119]	Direction and magnitude of any change in clozapine or olanzapine concentrations; no interaction study performed
Gastrointestinal tolerability	Dose-dependent nausea, vomiting, and delayed gastric emptying occur with retatrutide [27,102]	Clozapine-induced gastrointestinal hypomotility is a recognized, potentially fatal complication [104]	Whether combined exposure produces additive dysmotility risk; no data
Body composition	Substantial incretin-associated weight reduction can include loss of lean mass [80,81,82]; the incremental contribution of retatrutide’s glucagon component is uncertain	Elevated sarcopenic risk reported in schizophrenia [83]; lean-mass reduction should not itself be equated with sarcopenia [84]	Whether retatrutide causes functionally significant muscle loss in this population
Dysesthesia	Dysesthesia reported across the Phase 3 program; rates rose with dose in TRIUMPH-1, TRIUMPH-2, and TRIUMPH-4 but not in TRIUMPH-3 or TRANSCEND-T2D-1 [28,29,30,91]	Antipsychotic-treated patients may have pre-existing neurological or extrapyramidal symptoms that complicate attribution [118]	Mechanism unknown; whether it is distinguishable from antipsychotic-associated sensory symptoms is untested
Eating behavior and restrictive pathology	No retatrutide-specific eating-behavior data; GLP-1RAs alter appetite and eating behavior [31,107]	Case-level and pharmacovigilance evidence that GLP-1RAs can exacerbate or precipitate restrictive eating disorders [31,121]; eating disorders may be under-recognized in schizophrenia [123]	Whether retatrutide’s greater appetite suppression unmasks restrictive eating pathology in schizophrenia spectrum disorders, where detection is complicated by negative symptoms [123]

## Data Availability

No new data were created or analyzed in this study. Data sharing is not applicable to this article.

## Abbreviations

The following abbreviations are used in this manuscript:

LY3437943	Retatrutide (development code)
SGA	Second-Generation Antipsychotic
GLP-1RA	Glucagon-Like Peptide-1 Receptor Agonist
GLP-1	Glucagon-Like Peptide-1
GIP	Glucose-Dependent Insulinotropic Polypeptide
SMI	Severe Mental Illness
CATIE	Clinical Antipsychotic Trials of Intervention Effectiveness
NCEP	National Cholesterol Education Program
NHANES	National Health and Nutrition Examination Survey
RCT	Randomized Controlled Trial
AIWG	Antipsychotic-Induced Weight Gain
H1	Histamine H1
5-HT2C	5-Hydroxytryptamine (Serotonin) 2C
D2	Dopamine D2
M3	Muscarinic M3
AMPK	AMP-Activated Protein Kinase
POMC	Pro-Opiomelanocortin
BMI	Body Mass Index
FGA	First-Generation Antipsychotic
OR	Odds Ratio
CYP1A2	Cytochrome P450 1A2
CYP2C19	Cytochrome P450 2C19
DXA	Dual-Energy X-Ray Absorptiometry
ANGPTL3/8	Angiopoietin-Like Protein 3/8
LDL	Low-Density Lipoprotein
MASLD	Metabolic Dysfunction-Associated Steatotic Liver Disease
CNS	Central Nervous System
FDA	US Food and Drug Administration
PANSS	Positive and Negative Syndrome Scale
HbA1c	Glycated Hemoglobin
HDL	High-Density Lipoprotein
CI	Confidence Interval
CrI	Credible Interval
BACS	Brief Assessment of Cognition in Schizophrenia
HR	Hazard Ratio
MACE	Major Adverse Cardiovascular Events
GCG	Glucagon
T2D	Type 2 Diabetes
eGFR	Estimated Glomerular Filtration Rate
CYP3A4	Cytochrome P450 3A4
CYP2D6	Cytochrome P450 2D6
CYP	Cytochrome P450
UGT1A4	UDP-Glucuronosyltransferase 1A4
CIGH	Clozapine-Induced Gastrointestinal Hypomotility
MD	Mean Difference
